# Reciprocal regulation of the cholinic phenotype and epithelial-mesenchymal transition in glioblastoma cells

**DOI:** 10.18632/oncotarget.12337

**Published:** 2016-09-29

**Authors:** Katharina Koch, Rudolf Hartmann, Friederike Schröter, Abigail Kora Suwala, Donata Maciaczyk, Andrea Caroline Krüger, Dieter Willbold, Ulf Dietrich Kahlert, Jaroslaw Maciaczyk

**Affiliations:** ^1^ Neurosurgery Department, University Hospital Duesseldorf, Duesseldorf, Germany; ^2^ Institute of Complex Systems (ICS-6) Structural Biochemistry, Forschungszentrum Juelich, Juelich, Germany; ^3^ Institute for Stem Cell Research and Regenerative Medicine, Medical Faculty, Heinrich-Heine-University Duesseldorf, Duesseldorf, Germany; ^4^ Institut für Physikalische Biologie, Heinrich-Heine-University Duesseldorf, Duesseldorf, Germany; ^5^ Neurosurgery and Pediatric Neurosurgery, Medical University Lublin, Lublin, Poland

**Keywords:** glioblastoma, CSCs, epithelial-mesenchymal transition, choline metabolism, choline kinase alpha

## Abstract

Glioblastoma (GBM) is the most malignant brain tumor with very limited therapeutic options. Standard multimodal treatments, including surgical resection and combined radio-chemotherapy do not target the most aggressive subtype of glioma cells, brain tumor stem cells (BTSCs). BTSCs are thought to be responsible for tumor initiation, progression, and relapse. Furthermore, they have been associated with the expression of mesenchymal features as a result of epithelial-mesenchymal transition (EMT) thereby inducing tumor dissemination and chemo resistance. Using high resolution proton nuclear magnetic resonance spectroscopy (^1^H NMR) on GBM cell cultures we provide evidence that the expression of well-known EMT activators of the ZEB, TWIST and SNAI families and EMT target genes N-cadherin and VIMENTIN is associated with aberrant choline metabolism. The cholinic phenotype is characterized by high intracellular levels of phosphocholine and total choline derivatives and was associated with malignancy in various cancers. Both genetic and pharmacological inhibition of the cardinal choline metabolism regulator choline kinase alpha (CHKα) significantly reduces the cell viability, invasiveness, clonogenicity, and expression of EMT associated genes in GBM cells. Moreover, in some cell lines synergetic cytotoxic effects were observed when combining the standard of care chemotherapeutic temozolomide with the CHKα inhibitor V-11-0711. Taken together, specific inhibition of the enzymatic activity of CHKα is a powerful strategy to suppress EMT which opens the possibility to target chemo-resistant BTSCs through impairing their mesenchymal transdifferentiation. Moreover, the newly identified EMT-oncometabolic network may be helpful to monitor the invasive properties of glioblastomas and the success of anti-EMT therapy.

## INTRODUCTION

Glioblastoma (GBM) is the most prevalent primary malignant brain tumor with a median survival of less than two years. High levels of therapy resistance, strong cellular invasiveness and rapid cell growth demand aggressive multimodal therapies involving resection followed by radio-chemotherapy [1–3].

Recent evidence has pointed to the existence of brain tumor initiating cells in GBMs, so called brain tumor stem cells (BTSCs). This subpopulation of GBM cells with stem cell properties are considered to be the main cause of tumor development, progression and chemo-resistance also in malignant gliomas [4–7]. Recent studies described an important link between stem-like properties and the cells capacity to invade and disseminate into the microenvironment. This molecular switch called epithelial-mesenchymal transition (EMT) enables cell autonomous movement and extracellular matrix digestion, both cardinal features of tumor stem cells [8, 9]. EMT has been first described in epithelial tumors, but recent studies could link this phenomenon to tumor progression, invasion and therapy-resistance in GBM [10, 11]. EMT is precisely orchestrated by transcriptional modulators of the ZEB-, TWIST- and SNAI-families and results in expression of the mesenchymal markers N-cadherin and VIMENTIN [12–17].

Most recently EMT activation was associated with aberrant lipid metabolism [18], elucidating the importance of metabolic reprogramming for mesenchymal transformation. Multiple genetic and metabolic pathways are altered during malignant transformation, leading to extensive cellular growth and stress resistance. Thus, targeting onco-metabolic networks might be regarded as an innovative strategy to develop personalized cancer therapies [19, 20].

As such, cancer cells favor to generate ATP more rapidly through upregulation of glycolysis instead of oxidative phosphorylation. This so called Warburg effect is the best characterized metabolic phenotype in cancer [21]. Furthermore, cancer cells have been described to increase choline metabolism, detected by elevated intracellular levels of phosphocholine (PC) and total choline derivatives [tCho, glycerophosphocholine (GPC) + PC + free choline (fCho)]. The cardinal enzyme of choline metabolism, choline kinase alpha (CHKα), has been described as the main regulator of the cholinic phenotype and could be associated with tumor progression in various cancers [22–25].

Induction of the cholinic phenotype has been linked to malignant progression and aggressiveness in several cancers [26–28]. Here we suppressed EMT by blocking the potent EMT activator ZEB1 and analyzed the effect on the cellular metabolism of GBM cells using high resolution proton magnetic resonance spectroscopy (^1^H NMR). We identified a bidirectional link between EMT and choline metabolism and revealed that targeting CHKα is a powerful strategy to suppress EMT status and BTSC properties of GBM cells. Moreover, metabolites that are connected to EMT progression can be monitored with *ex vivo* imaging technology and therefore have strong potential for rapid clinical translation in tumor diagnostics and surveillance.

## RESULTS

### ZEB1 knockdown reduces the viability of GBM cells

**Figure 1:**
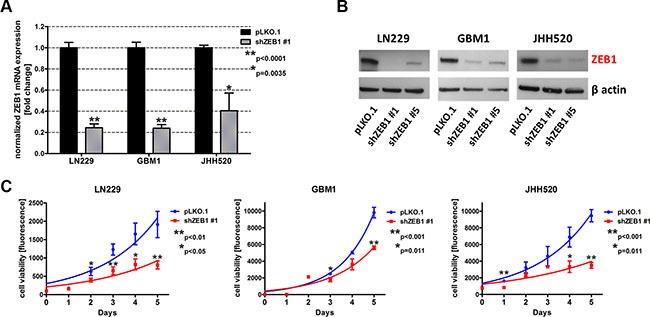
ZEB1 knockdown reduces the cell viability GBM cell lines (LN229, GBM1 and JHH520) were transduced with lentiviral particles containing shZEB1 plasmids and knockdown efficiency was confirmed using RT qPCR (**A**) and Western blotting (**B**). (**C**) The cell viability of ZEB1 knockdown cells was reduced as compared to control (pLKO.1) cells. Exponential growth curves were calculated for each condition and displayed in the graphs. The data is represented as mean ± SD (*n* = 3).

In order to analyze whether epithelial to mesenchymal transition (EMT) affects metabolic pathways in GBMs, we established stable tumor models with suppressed expression of the core EMT activator ZEB1 in three GBM cell lines (LN229, GBM1 and JHH520) through RNA interference technology. The knockdown efficiency was confirmed on mRNA and protein level. RT qPCR results showed that transduction with either shZEB1 #1 or shZEB1 #5 resulted in a significant reduction of *ZEB1* mRNA by 60%–80% (Figure 1A, shown for shZEB1 #1). Western blotting confirmed the efficacy of both shZEB1 shRNAs, leading to a distinctive reduction of ZEB1 protein levels (Figure 1B).

Previous research of our group revealed the role of ZEB1 in invasion of GBM cells [10, 12]. To further investigate the phenotype of ZEB1 depletion, we analyzed the cell viability after transduction with shZEB1 #1 or control vector. Therefore, we performed the TiterBlue^®^ viability assay with LN229, GBM1, and JHH520 shZEB1 #1 or control cells over five consecutive days. Figure 1C shows that ZEB1 knockdown decreases the viability of all three tested GBM cell lines.

### ZEB1 knockdown alters the cellular metabolism of GBM cells

**Figure 2:**
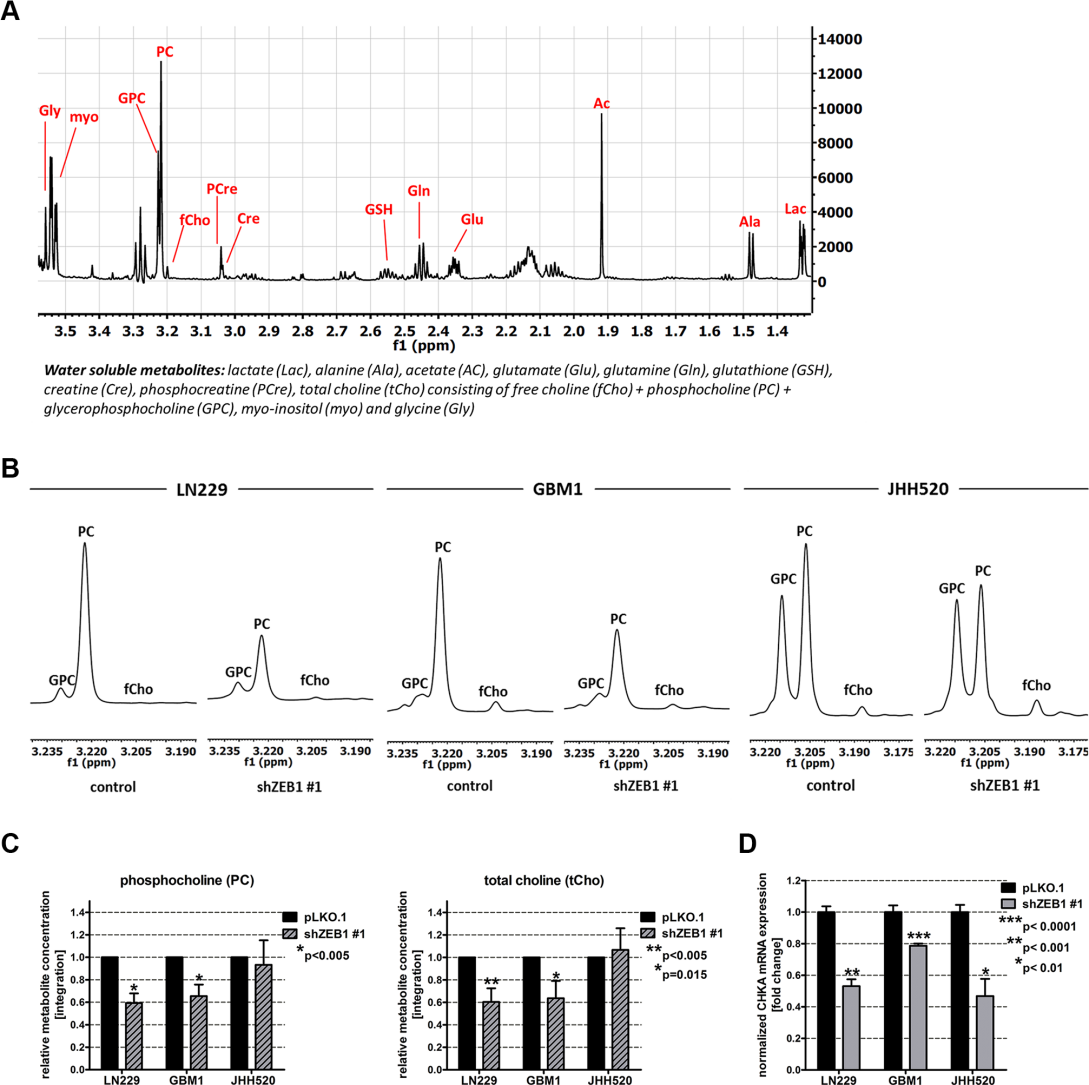
EMT reduction by ZEB1 knockdown alters choline metabolism (**A**) Overview of a ^1^H-NMR spectrum of metabolic extracts of GBM cells. (**B**) Expanded regions of ^1^H-NMR spectra of control and shZEB1 transduced cells showing the main choline metabolites. (**C**) Quantitation of ^1^H-NMR spectra for PC and tCho from metabolic extracts of ZEB1 knockdown and control cells. (**D**) Expression of *CHKΑ* mRNA in ZEB1 knockdown cells was measured using RT qPCR and compared to pLKO.1 transduced cells. Abbreviations: ppm, parts per million. The data is represented as mean ± SD (*n* = 3).

In order to assess whether the reduction of EMT influences the metabolism of GBM cells, we extracted water-soluble metabolites from cells with ZEB1 suppression and control cells. The extracts were analyzed via ^1^H NMR spectroscopy and differences in the relative metabolite concentrations of both conditions were calculated. Figure 2A shows a typical spectrum of GBM cell metabolic extracts with the most prominent peaks representing lactate (Lac), alanine (Ala), acetate (Ac), glutamate (Glu), glutamine (Gln), glutathione (GSH), creatine (Cre), phosphocreatine (PCre), free choline (fCho), phosphocholine (PC), glycerophosphocholine (GPC), total choline (tCho; comprising fCho, PC and GPC), myo-inositol (myo), and glycine (Gly). ZEB1 knockdown significantly (*p* < 0.05) alters the intracellular levels of multiple metabolites belonging to various metabolic networks including Glu, GSH, Cre, PC, tCho, and Gly (Supplementary Figure S1). Given the importance of choline metabolism in malignant transformation and its utility for clinical brain tumor diagnostics [29] we decided to focus our studies on alterations in choline derivatives.

### The EMT activator ZEB1 alters choline metabolism by regulating choline kinase alpha (CHKα)

ZEB1 depletion reduced the cholinic phenotype, since we detected decreased amounts of the choline metabolites PC and tCho in ZEB1 knockdown cells. Representative choline metabolite peaks of ^1^H NMR spectra and corresponding relative quantifications are shown in Figure 2B and 2C, respectively. ZEB1 knockdown led to a significant reduction of PC in LN229 (*p* < 0.01) and GBM1 cells (*p* < 0.01). Furthermore, we could detect a significant reduction of tCho (*p* < 0.01 for LN229 and *p* = 0.015 for GBM1) concentrations. In JHH520 GBM cells, ZEB1 depletion did not significantly change PC or tCho concentrations. Next we wanted to investigate which metabolic regulator might account for the ZEB1-mediated alterations in choline metabolism and investigated the expression of the cardinal choline metabolism regulating enzyme CHKα. Strikingly, ZEB1 inhibition resulted in suppressed *CHKΑ* mRNA expression in all tested cell lines (*p* < 0.001 for LN229, *p* < 0.0001 for GBM1, and *p* < 0.01 for JHH520 cells) (Figure 2D). As CHKα phosphorylates free choline to generate PC, we speculate that a reduction of CHKα activity most likely causes the decrease of PC and tCho that we observed after ZEB1 knockdown. This initial observation of a putative ZEB1-CHKα link let us investigate whether it is a bidirectionally regulated loop and if targeted CHKα inhibition may impact the EMT properties of GBM cells.

### CHKα knockdown alters choline metabolism similar to ZEB1 suppression

**Figure 3:**
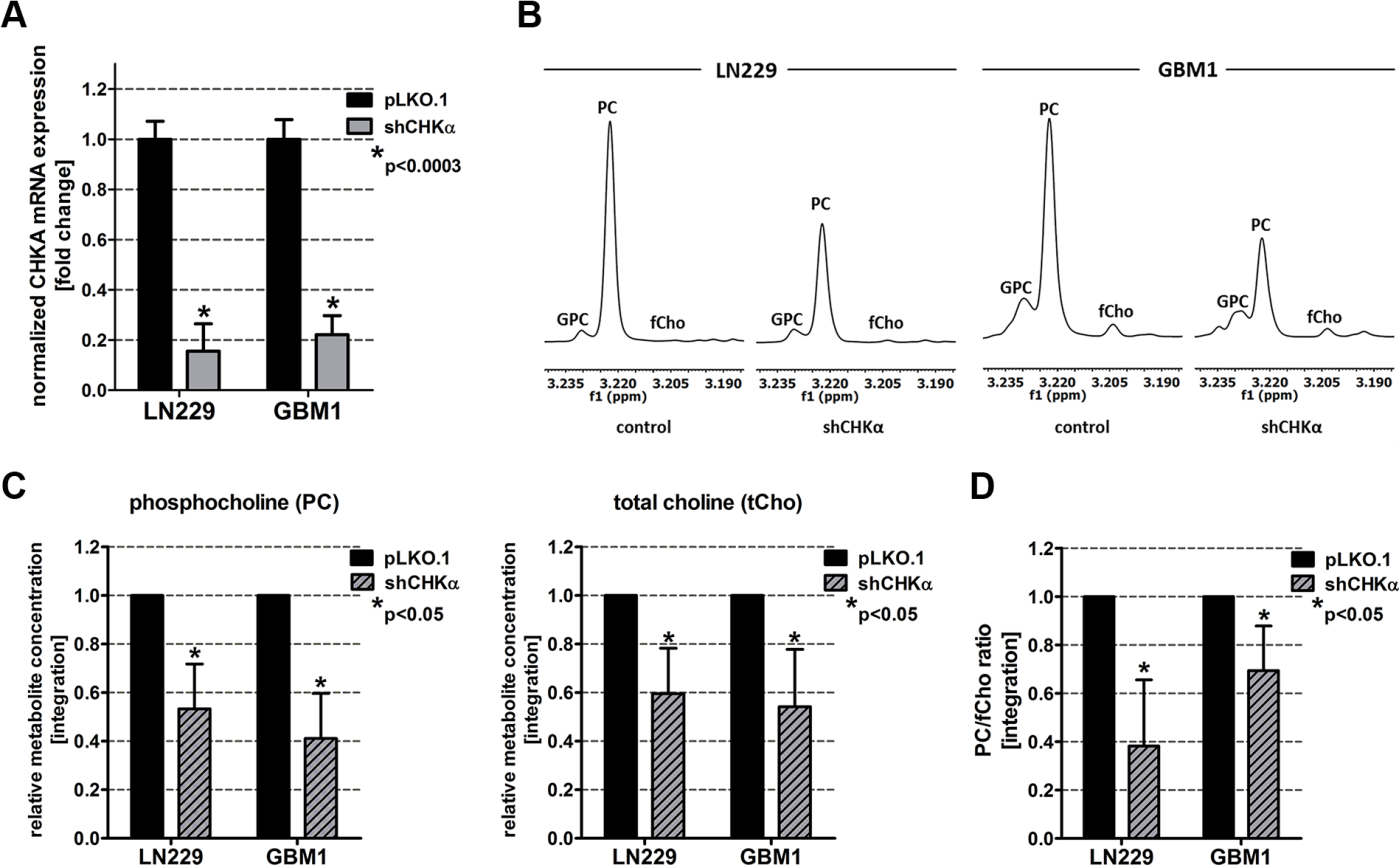
Choline Kinase alpha (CHKα) knockdown leads to similar alterations in choline metabolism as ZEB1 knockdown (**A**) CHKα suppression was confirmed on mRNA level by RT qPCR. (**B**) Expanded regions of ^1^H-NMR spectra of control and shCHKα transduced LN229 and GBM1 cells showing the main choline metabolites. (**C**) Quantitation of ^1^H-NMR spectra for PC and tCho from metabolic extracts of CHKα knockdown and control cells. (**D**) Ratio of phosphocholine and free choline (product and educt of CHKα). The data is represented as mean ± SD (*n* = 3).

In order to test the influence of CHKα inhibition on EMT in GBM cells, we performed a genetic CHKα knockdown using short hairpin interference technology (shRNAs). RT qPCR analysis revealed a significant (*p* < 0.0003) reduction of CHKα gene expression of up to 75% (Figure 3A). ^1^H NMR analysis of metabolic extracts showed alterations in relative choline metabolite concentrations in shCHKα cells similar to those found after ZEB1 knockdown. In concordance, the PC signal at 3.22 ppm was highly reduced after CHKα depletion (Figure 3B). Further statistical analysis highlighted a significant reduction of PC (*p* < 0.05) and tCho (*p* < 0.05) in CHKα knockdown cells as compared to control vector transduced cells (Figure 3C). In addition, CHKα knockdown significantly (*p* < 0.05) decreased the product (PC) to educt (fCho) ratio of CHKα, suggesting that the reduced amount of PC results from CHKα suppression (Figure 3D). We also noticed alterations in intracellular concentrations of other metabolites after CHKα depletion as presented in Supplementary Figure S2A. CHKα knockdown significantly increased Cre (*p* < 0.05) and GPC (*p* < 0.05) and decreased Lac (*p* < 0.05). We further detected a significant increase of the Cre/PCre ratio in both LN229 (*p* < 0.05) and GBM1 (*p* < 0.05) shCHKα cells (Supplementary Figure S2B), indicating reduced phosphorylation of Cre by creatine kinase brain-type (CKB). Indeed, we could confirm decreased expression of *CKB* in LN229 (*p* < 0.05) and GBM1 (*p* > 0.05) shCHKα cells (Supplementary Figure S2C).

### Cells that express CHKα are positive for the stem cell marker SOX2 and the mesenchymal marker VIMENTIN

**Figure 4:**
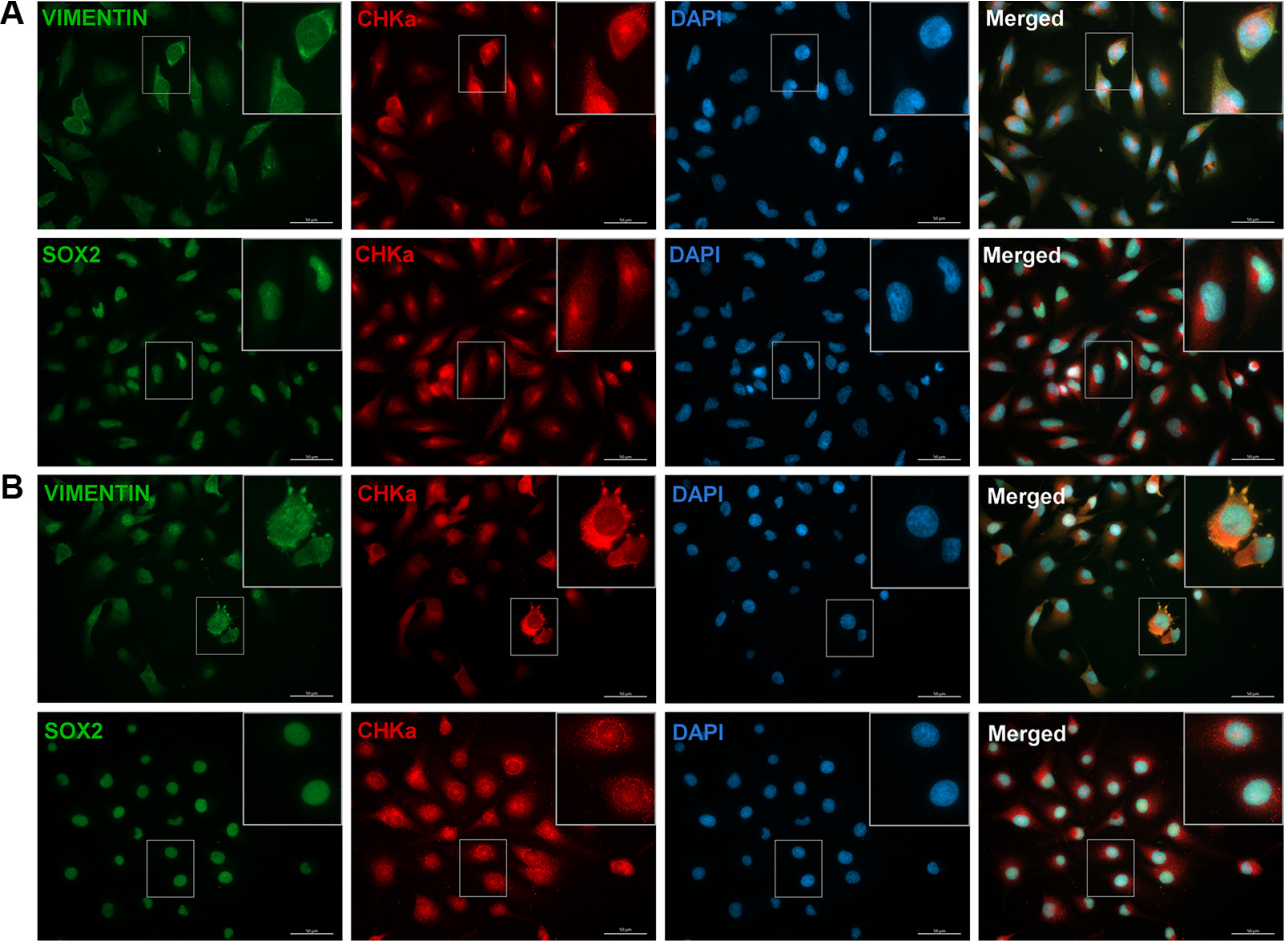
CHKα is co-expressed with the mesenchymal marker VIMENTIN in GBM cells Immunocytochemical staining was performed for CHKα (red), VIMENTIN (green), SOX2 (green), and DAPI (blue) in LN229 (**A**) and JHH520 (**B**) cells. All cells were stained positive for the three tested proteins. VIMENTIN/CHKα/DAPI and SOX2/CHKα/DAPI co-stainings revealed that CHKα expression correlates with the expression of VIMENTIN but not with SOX2 in all tested cell lines. Scale bar: 50 μm; GBM1 not shown.

As we found a link between CHKα and the EMT activator ZEB1, we further analyzed if cells with CHKα also express other EMT/BTSC markers. We therefore performed fluorescence microscopy on all analyzed cell lines and stained for the mesenchymal marker VIMENTIN and the stem cell marker SOX2 in combination with CHKα (Figure 4). CHKα staining could be detected in the cytoplasm as well as in the nucleus of the whole cell population, although the expression level differed between cells. The transcription factor SOX2 was expressed in the nucleus of all cells, elucidating the immature character of GBM cells. VIMENTIN could be detected predominantly in the cytoplasm and the expression level differed between the cells. Most interestingly, cells with more VIMENTIN staining tend to have higher expression of CHKα. In the co-staining for SOX2 and CHKα we could not detect coherences, as all cells exhibit a strong SOX2 staining. Of note, especially in LN229 cells CHKα tends to accumulate in an area close to the nucleus in a puncta-like structure, presumably in cells undergoing cell division (Figure 4A). In the past, several publications could correlate CHKα expression with cell cycle regulation and mitosis in different tumor entities [30, 31]. Our results now suggest that there could be a correlation between cell division and CHKα expression in GBM as well.

### Choline kinase alpha knockdown reduces the expression of EMT activators and neural stem cell markers

**Figure 5:**
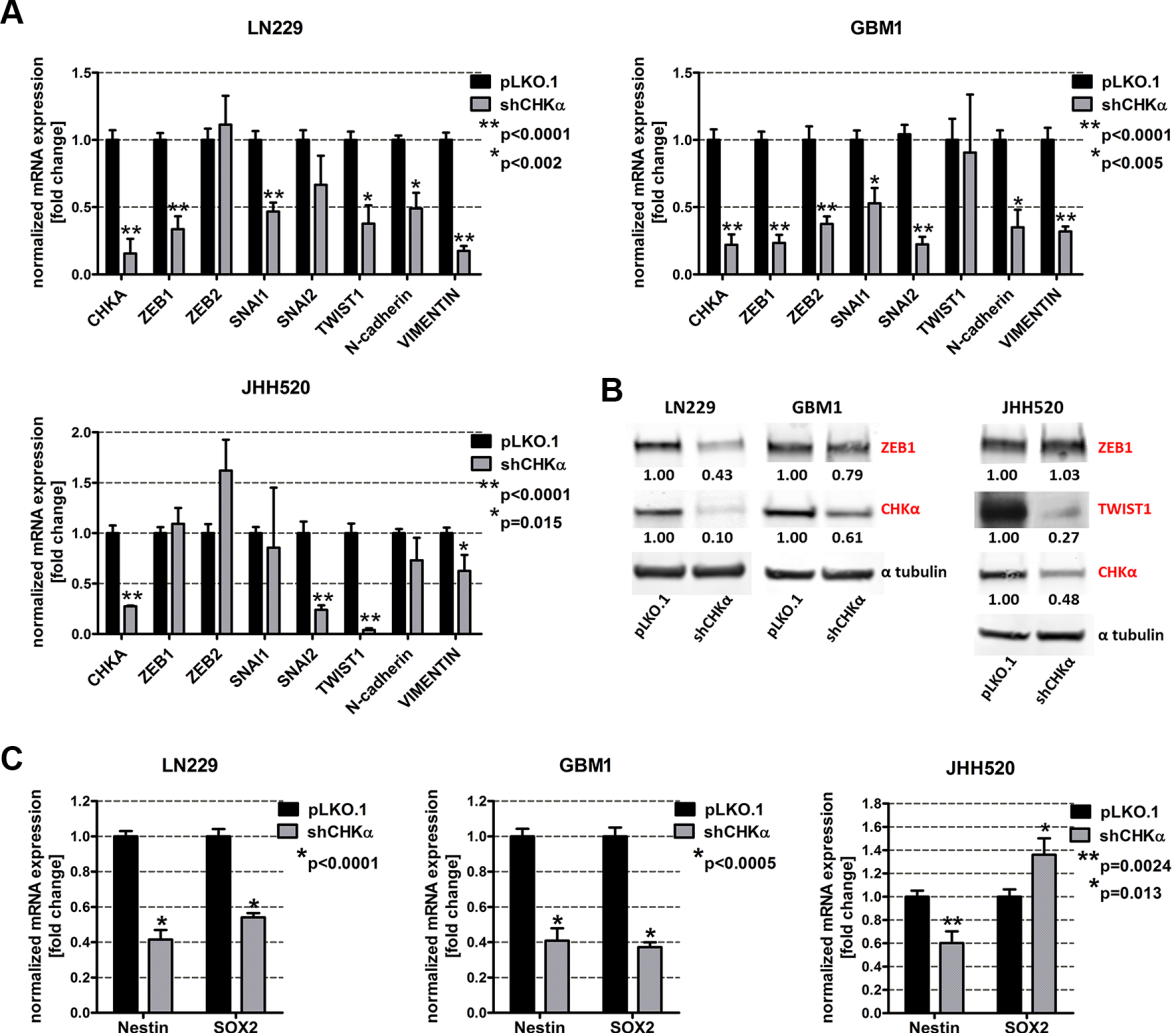
Suppression of CHKα reduces the expression of EMT-associated genes and neural stem cell markers (**A**) *ZEB1, ZEB2, SNAI1, SNAI2*, *TWIST1*, *N-cadherin*, and *VIMENTIN* mRNA levels were analyzed by RT qPCR in shCHKα cells and compared to controls. (**B**) ZEB1, TWIST1, and CHKα protein expression levels were detected using immunoblotting in shCHKα and control cells. (**C**) *Nestin* and *SOX2* mRNA expression levels in shCHKα cells were analyzed by RT qPCR and compared to control cells. The data is represented as mean ± SD (*n* = 3).

Given our results of a putative EMT-choline metabolism loop and the co-expression of CHKα and the mesenchymal marker VIMENTIN, we tested the influence of CHKα suppression on the expression of EMT associated genes. We found that suppression of CHKα caused a strong reduction of ZEB1, ZEB2, TWIST 1, SNAI1, and SNAI2. Quantification of RT qPCR results revealed a 66% reduction of *ZEB1* (*p* < 0.001), 62% reduction of *TWIST1* (*p* = 0.0014) and a 53% reduction of *SNAI1* (*p* < 0.001) in LN229 and a 77% reduction of *ZEB1* (*p* < 0.0001), a 63% reduction of *ZEB2* (*p* < 0.0001), a 47% reduction of *SNAI1* (*p* = 0.002), and a 78% reduction of *SNAI2* in GBM1 cells (Figure 5A). ZEB1 suppression was further confirmed on protein level with up to 57% reduction in LN229 and 21% reduction in GBM1 cells (Figure 5B). CHKα depletion in JHH520 cells suppressed SNAI2 mRNA expression by 76%, *TWIST1* mRNA expression by 96% (*p* < 0.0001) and TWIST1 protein expression by up to 73% but had no effect on ZEB expression. TWIST1 baseline protein expression in LN229 and GBM1 was too low to be detected. Furthermore, we assessed the influence of CHKα on the expression of known EMT target genes and found that CHKα depletion reduced the expression of N-cadherin by 50% in LN229 (*p* < 0.02) and by 65% in GBM1 (*p* < 0.05) cells. The expression of *VIMENTIN* was reduced by 83% in LN229 (*p* < 0.0001), by 68% in GBM1 (*p* < 0.0001), and by 38% in JHH520 (*p* < 0.02) cells with CHKα knockdown, which is in concordance with our immunofluorescence data. As mesenchymal cells are characterized by high levels of stem cells markers, we analyzed the expression of the neural stem cell markers Nestin and SOX2 in shCHKα and control cells. CHKα suppression significantly reduced the expression of *NESTIN* in LN229 (*p* < 0.0001), GBM1 (*p* < 0.0001) and JHH520 (*p* = 0.0024) cells and *SOX2* in LN229 (*p* < 0.0001) and GBM1 (*p* < 0.0001) cells (Figure 5C). Together these results provide important insight into the ability of CHKα to regulate the activation of EMT and the stem cell character of GBM cells.

### Knockdown of choline kinase alpha reduces the cellular viability, invasiveness and clonogenicity

**Figure 6:**
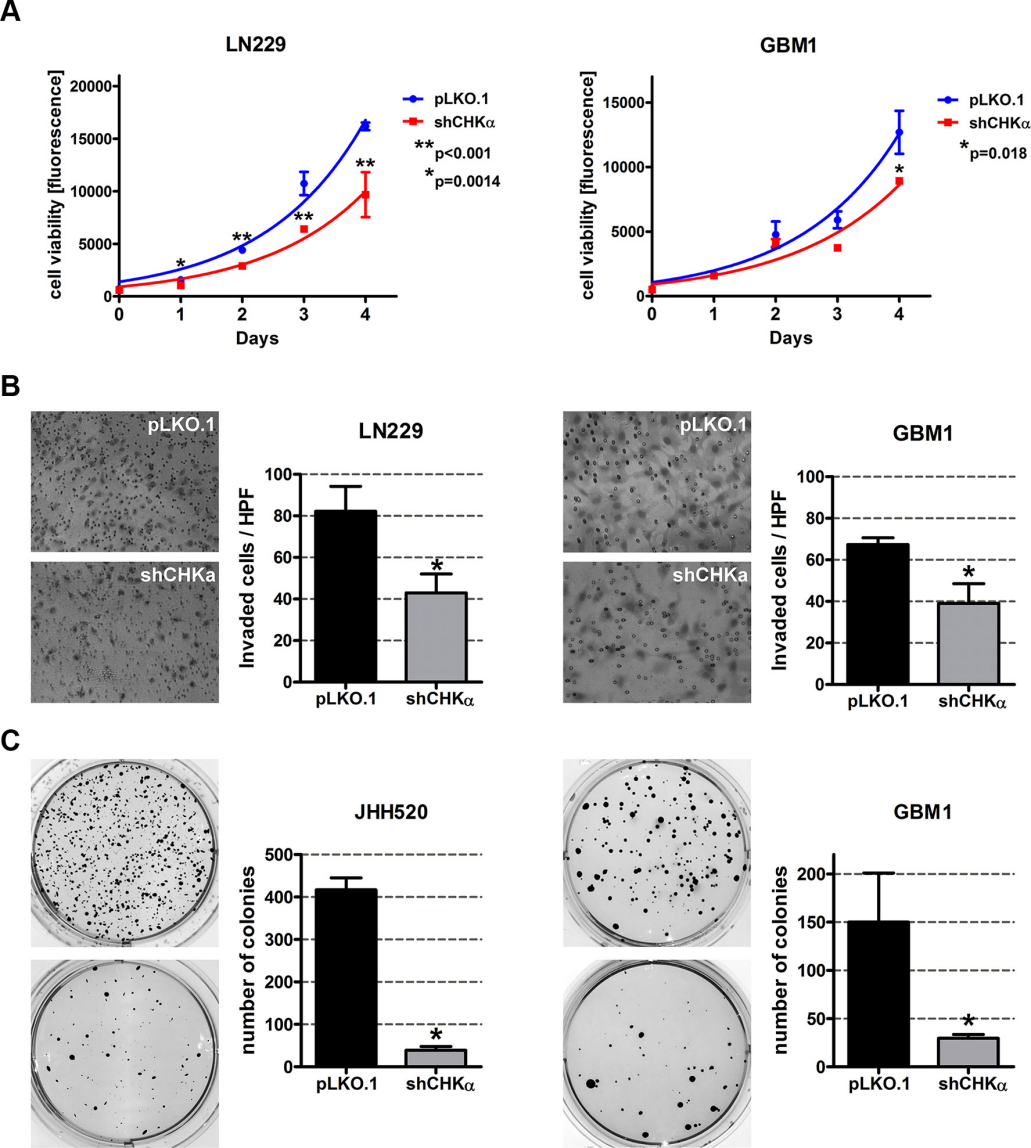
CHKα knockdown reduces the viability, invasiveness and clonogenicity of GBM cells (**A**) Knockdown of CHKα reduces the viability of LN229 and GBM1 cells. Exponential growth curves were calculated for each condition and displayed in the graphs. (**B**) Cells with impaired CHKα expression are less invasive as assessed with modified Boyden chamber assays for 24 h. Representative pictures of hematoxylin stained invaded cells and quantifications of three Boyden chamber experiments are shown (*p < 0.05). (**C**) CHKα suppression reduces the clonogenicity of JHH520 and GBM1 cells as assessed by soft agar assays. Representative pictures of NBT stained colonies and quantifications of three colony forming assays are shown (*p < 0.05). Abbreviations: HPF, high power field; NBT, 4-Nitro blue tetrazolium chloride. The data is represented as mean ± SD (*n* = 3).

Next we investigated if CHKα suppression is able to reduce the mesenchymal phenotype of GBM cells. Compared to their control counterparts, CHKα knockdown cells exhibit significantly reduced viability (Figure 5A). Furthermore, we assessed the invasive behavior after CHKα inhibition with modified Boyden chamber assays and found a 48% decrease in invading cells for LN229 (*p* = 0.011) and a 42% decrease for GBM1 (*p* = 0.0082) cells (Figure 6B). Moreover, depletion of CHKα significantly diminished the anchorage independent *in vitro* clonogenicity of our tested neurospheres lines (by 90% in JHH520, *p* < 0.0001 and by 80% in GBM1, *p* < 0.05). Taken together, shCHKα cells exhibit fewer properties of mesenchymal cells than control vector transduced cells. Furthermore, we looked at RNA sequencing data of different tumor compartments which were identified in the Anatomic Structures ISH Survey and isolated by laser microdissection. Increased expression of CHKα could be found in the infiltrative region and the leading tumor edge of GBMs (Supplementary Figure S3).

### Pharmacological inhibition of choline kinase alpha reduces the expression of the EMT activators ZEB1 and TWIST1 in GBM cells

Targeted suppression of EMT in cancer cells is of highest clinical interest as successful mesenchymal reprogramming appears to be crucial for the invasive properties of a majority of malignant cells. In a translational approach, we applied the CHKα-inhibitor V-11-0711 (Vertex Pharmaceuticals Incorporated) on GBM cells and tested subsequent alterations in geno- and phenotype. V-11-0711 has been shown to specifically suppress CHKα catalytic activity in breast cancer and HeLa cells [32, 33].

**Figure 7:**
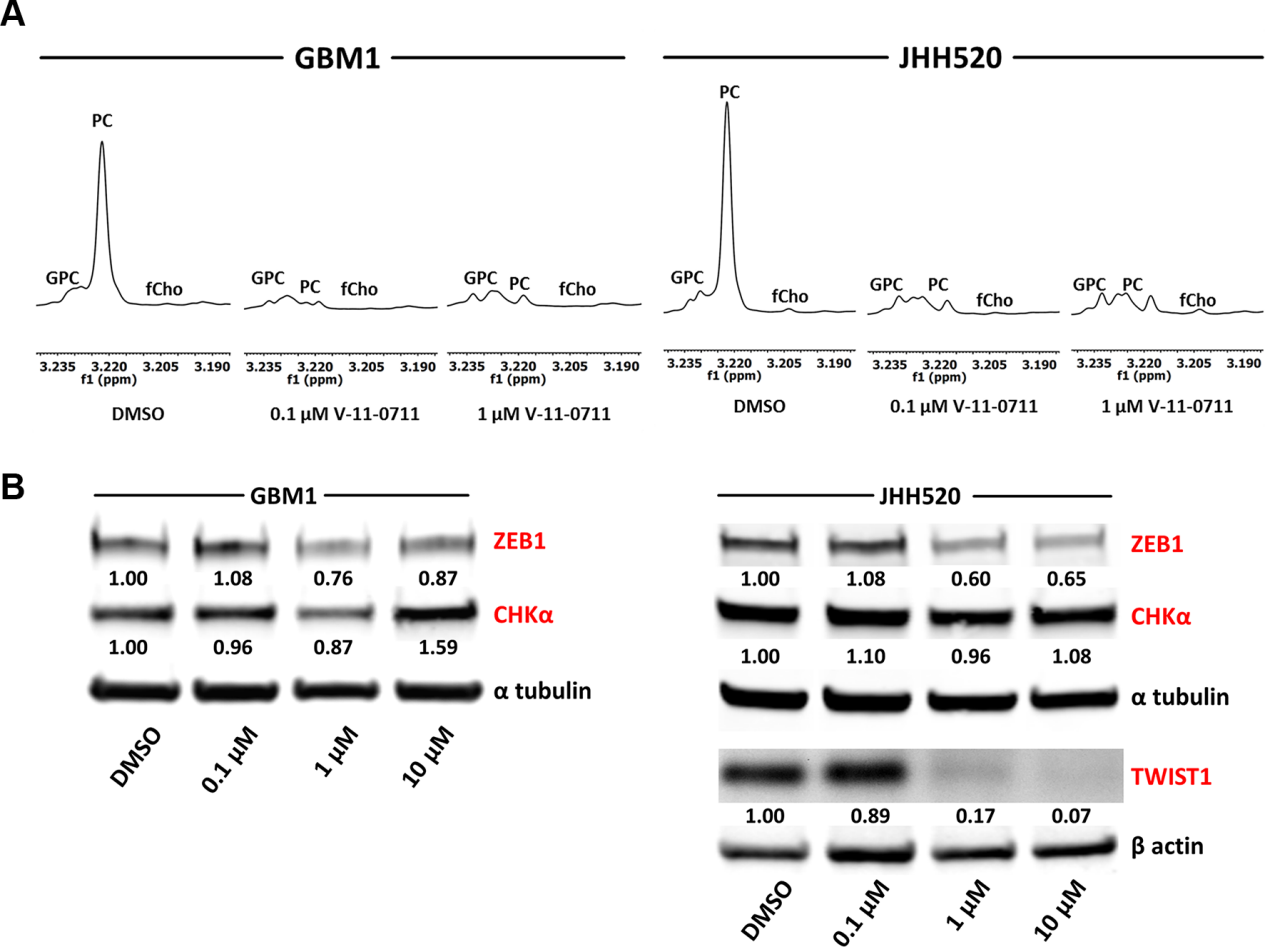
Treatment with the choline kinase inhibitor V-11-0711 alters choline metabolism and reduces the expression of the EMT activators ZEB1 and TWIST1 in GBM cells (**A**) Expanded regions of ^1^H-NMR spectra for the main choline metabolites of DMSO and V-11-0711 treated cells show the effectiveness of V-11-0711 treatment (**B**) V-11-0711 treatment led to a suppression of ZEB1 and induction of CHKα protein in GBM1 and a suppression of both ZEB1 and TWIST1 protein in JHH520 cells as measured by immunoblotting. Alpha tubulin and beta actin immunoblotting were used as loading controls. Abbreviations: DMSO, dimethyl sulfoxide. The data is represented as mean ± SD (*n* = 3).

GBM1 and JHH520 neurospheres were treated with DMSO, 0.1 μM V-11-0711 or 1 μM V-11-0711 for 48 h and metabolic extracts were analyzed by ^1^H NMR spectroscopy. As the PC signal at 3.22 ppm was highly reduced in the drug treated cells we confirmed the ability of V-11-0711 to inhibit the enzymatic activity of CHKα in GBM cells (Figure 7A).

Strikingly, pharmacological suppression of CHKα further led to a dose dependent reduction of ZEB1 protein in GBM1 and both ZEB1 and TWIST1 protein in JHH520 cells (Figure 7B). Additionally, V-11-0711 induced a dose dependent increase of CHKα protein in GBM1 but not in JHH520 cell line.

### Pharmacological inhibition of choline kinase alpha reduces the viability, invasiveness and clonogenicity of GBM cells

**Figure 8:**
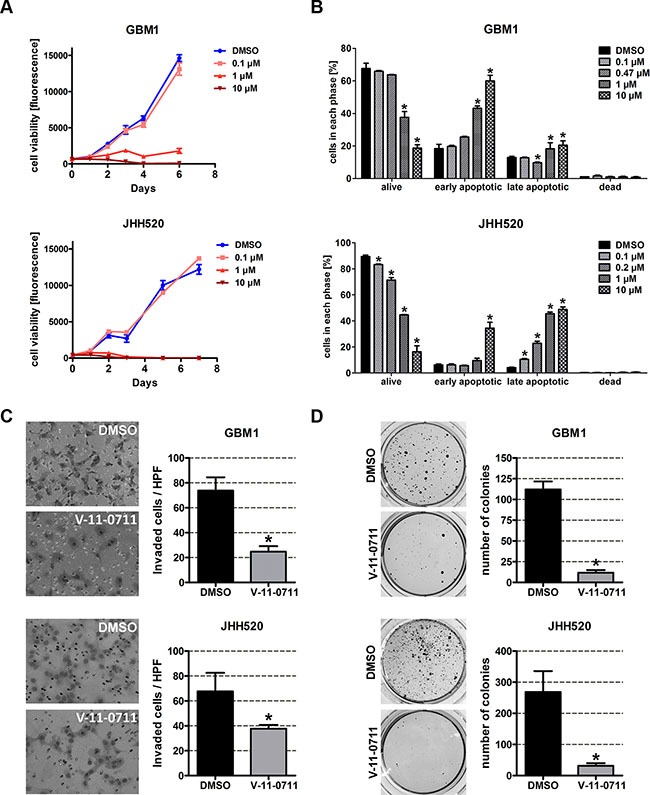
V-11-0711 treatment reduces the viability, invasiveness, and clonogenicity of GBM cells (**A**) Cell viability was impaired by V-11-0711 treatment in a dose dependent manner. Exponential growth curves were calculated for each condition and displayed in the graphs. (**B**) V-11-0711 induces apoptosis as assessed with the Muse^®^ Annexin V and Dead Cell Kit. Pharmacological inhibition of CHKα with 0.47 μM (GBM1) and 0.2 μM (JHH520) V-11-0711 led to diminished invasive capacity (**C**) of GBM1 and JHH520 cells (*p < 0.05) and decreased the *in vitro* clonogenicity (*p < 0.005) (**D**). Abbreviations: DMSO, dimethyl sulfoxide; NBT, 4-Nitro blue tetrazolium chloride. The data is represented as mean ± SD (*n* = 3).

Next we wanted to prove that pharmacological inhibition of CHKα can resemble the effect of our genetic inhibition model. Strikingly, treatment with 1 μM or 10 μM V-11-0711 for 48 h drastically reduced the cell viability of GBM1 and JHH520 cells (Figure 8A) which we could associate with dose-dependent induction of apoptosis (Figure 8B). For the following *in vitro* invasion and clonogenicity assays we therefore used V-11-0711 concentrations which induce no (0.47 μM for GBM1) or only mild apoptosis (0.2 μM for JHH520). We performed modified Boyden chamber transwell assays for 24 h in the presence of either V-11-0711 or DMSO. The cells were preincubated with indicated concentrations of the drug for 24 h. Two representative pictures and statistical analysis of three Boyden chamber assays are shown in Figure 8C. We could detect a 66.5% decrease of invading cells in GBM1 (*p* = 0.0018) and a 44.3% decrease in JHH520 (*p* = 0.027) cells after drug exposure as compared to controls. Moreover, the capacity of the cells to form colonies was reduced significantly by 90% in GBM1 (*p* < 0.0001) and by 88% in JHH520 (*p* < 0.005) cells following treatment with V 11-0711. In summary, our results unequivocally prove the potential of CHKα inhibition to precisely target the invasive and clonogenic population of GBM cells by suppressing key EMT activators and EMT target genes.

### Combination treatment with temozolomide and V-11-0711 has synergistic effects in a subset of GBM cell lines

**Figure 9:**
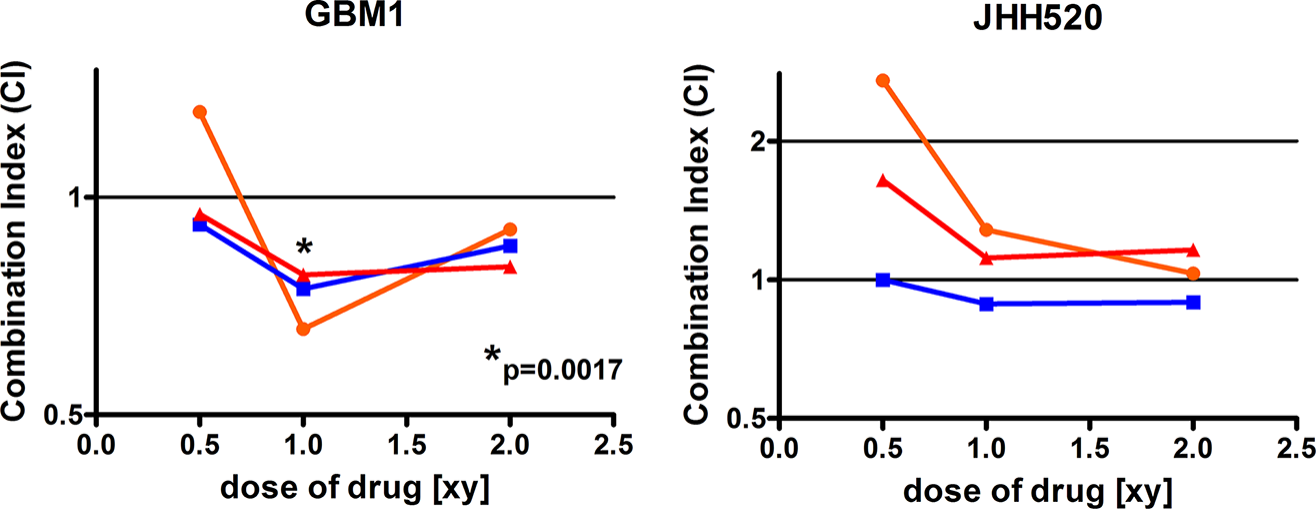
Combinatory treatment with Temozolomide and V-11-0711 has synergistic effects in GBM1 cells V-11-0711 in combination with TMZ led constantly to synergistic cytotoxic effects in GBM1 but not in JHH520 cells (*p < 0.05). Abbreviations: DMSO, dimethyl sulfoxide; CI, Combination Index (CI < 1, synergistic; CI = 1, additive; CI >1, antagonistic); [xy], multiple of the single TMZ (x) and V-11-0711 (y) dose; Single TMZ dose (x): 17.5 μM for GBM1 and JHH520; Single V-11-0711 doses (y): 0.47 μM for GBM1 and 0.2 μM for JHH520.

ZEB1 has been associated with chemo resistance in GBMs [11]. In order to assess if CHKα inhibition induces sensitivity to the standard chemotherapeutic in GBM therapy, temozolomide (TMZ), GBM1 and JHH520 cells were treated with either single drugs or drug combinations of V-11-0711 and TMZ. Interestingly, treatment of GBM1 cells with V-11-0711 and TMZ together had synergistic anti-growth effects in all tested drug combinations (correlation index < 1) (Figure 9), whereas for JHH520 we could not detect constant synergy.

## DISCUSSION

In this study we could show for the first time that EMT can be reduced by targeting choline metabolism in GBM. Both genetic reduction through RNA interference and pharmacological inhibition of CHKα with the small molecule inhibitor V-11-0711 in GBM cells significantly reduced the expression of EMT activators and EMT target genes. Furthermore, we observed impaired cellular invasiveness, clonogenicity, viability as well as reduced expression of neural stem cell markers, all hallmarks of EMT [12], upon interfering with choline metabolism.

Our approach was to study whether EMT is associated with metabolic processes in GBM cells. We therefore used a previously described EMT inhibition model based on knockdown of ZEB1 [12]. In our model we observed decreased cell viability and changes in various intracellular metabolite concentrations in cells with reduced ZEB1. The role of ZEB1 in cell proliferation and tumor growth is not fully understood. A hallmark paper investigating the role of ZEB1 in gliomagenesis reported doubling of proliferation upon ZEB1 impairment [11], although changes in ZEB1 caused by activation of WNT signaling had no effect on proliferation [10]. Similarly, contrary results are published in other systems showing that ZEB1 promotes [34, 35], impairs [36, 37], or has no effect [38] on proliferation and cellular growth. On the metabolite level we observed a significant decrease of PC and tCho in LN229 and GBM1 cells (Figure 2). High concentrations of PC and tCho, also referred to as the cholinic phenotype, could be correlated with malignant transformation in various types of cancers [27, 28], thus our results hint at reduced malignancy after EMT inhibition. We found that the molecular cause for the reduced cholinic phenotype is impaired CHKα expression in cells with EMT suppression, both confirmed on mRNA and protein level. Also in breast cancer, CHKα activity has been described as the main regulator of the cholinic phenotype [22]. CHKα catalyzes the phosphorylation of free choline to PC during the synthesis of the membrane lipid phosphatidylcholine, thus CHKα is of highest importance for the integrity of the cell membrane and a variety of signaling pathways involving membrane lipids and membrane anchored proteins [39, 40]. As expected, direct suppression of CHKα through RNA-interference reduced the cholinic phenotype to levels similar or even lower than observed after ZEB1 suppression (Figure 3). Taken together, these results suggest that the activity of CHKα is associated with EMT in GBM cells. CHKα is broadly expressed in our tested cell line models (Figure 4). This is not surprising as CHKα catalyzes important steps during membrane lipid synthesis and thus appears to be indispensable for the cell survival and proper function. Our co-labeling experiments revealed that cells with high levels of CHKα express high levels of the EMT target gene VIMENTIN [15–17]. Furthermore, we detected strong expression of the neural and glioma stem cell marker SOX2 [41, 42] in all our tested models. We could not detect any differences in SOX2 expression in cells with high or low levels of CHKα.

Interestingly, CHKα knockdown reduced the protein expression of the EMT activator ZEB1 in LN229 and GBM1 cells and TWIST1 in JHH520 cells (Figure 5). These results indicate that there is a bidirectional link between EMT and choline metabolism in GBM and that CHKα regulates EMT in a global way affecting the expression of several EMT activators. This hypothesis was further corroborated by the reduced *ZEB1*, *ZEB2*, *SNAI1*, *SNAI2 and TWIST1* mRNA levels as well as lowered expression of EMT targets N-cadherin and VIMENTIN after CHKα knockdown [15–17]. Probably as a result of the reduced expression of EMT associated genes, cells with CHKα suppression exhibit fewer phenotypic properties of mesenchymal cells, since we detected significantly reduced invasiveness and clonogenicity in shCHKα cells (Figure 6). Concisely, ZEB1, ZEB2, and TWIST1 have all been described to promote invasion, and clonogenicity in GBM and other tumors [12, 43–48]. Additionally we found increased CHKα expression on the infiltrative leading edge of GBMs as compared to tumor parenchyma, indicating CHKα expression is associated with invasive properties of the tumor cells in GBM patients (Supplementary Figure S3). Taken together, this led us hypothesize that CHKα suppression is able to suppress the EMT phenotype in GBM. Of note, previous studies with breast cancer and ovarian cancer cells correlated CHKα expression with increased cellular invasiveness, migration, and proliferation [23, 49], indicating the importance of CHKα as a regulator of the mesenchymal phenotype in other tumors than GBM.

EMT has been associated with cancer stemness in a variety of tumors [8, 9]. Along with the impaired *in vitro* clonogenicity after CHKα blockade, we observed decreased expression of the stem cell genes Nestin and SOX2 after CHKα suppression. Both genes are established BTSC markers with prognostic value and clinical relevance [41, 50].

Mesenchymal transformation is crucial for the generation of chemo- and radioresistant BTSCs in malignant brain tumors [51, 52], thus identifying achievable approaches to inhibit EMT is of highest clinical interest. Given our evidences from the genetic studies that CHKα controls mesenchymal differentiation, we tested the effect of compound based CHKα inhibition using the CHKα inhibitor V-11-0711 which recently has been shown to selectively inhibit CHKα activity [32, 33]. Analysis of metabolic extracts with ^1^H NMR spectroscopy proved the enzyme inhibiting capability of V-11-0711 in GBM cells as we did not detect any CHKα product - PC at 3.22 ppm – after drug exposure (Figure 7). Strikingly, V-11-0711 treatment reduced the expression of the EMT activator ZEB1 in GBM1 and both ZEB1 and TWIST1 in JHH520 cells in a dose-dependent fashion. Furthermore, we detected a significant reduction in cellular viability, survival, invasiveness, and clonogenicity after drug treatment, confirming our data with genetic CHKα inhibition. Since V-11-0711 dose dependently induces apoptosis in GBM cells we used drug concentrations for the invasion and colony forming assays inducing no (GBM1) or very mild (JHH520) apoptosis. We further detected a dose dependent increase of the CHKα protein level in GBM1 cells, indicating a rescue mechanism probably triggered by the dramatically decreased PC concentrations. This also suggests that not the protein level but rather the enzymatic activity of CHKα is crucial for EMT regulation in GBM. Therefore, CHKα suppression could be a novel approach to precisely target highly invasive cancer stem cells. Of note, comprehensive CHKα inhibition through systemic administration of V-11-0711 or other inhibitors will impair the cell membrane synthesis in all cells and phenotypic effects will not be limited to the cancerous lesions. Therefore we envision that this potential form of therapy could be applicable as local therapy in the open resection cavity or continuous through convection enhanced delivery through intratumoraly implanted catheters to target the disseminated single cells in the next vicinity. Another possibility could be highly selective intra-arterial transfer of siRNAs or shRNAs through cerebral circulation over the blood brain barrier (BBB) to the side of the tumor to suppress CHKα by means of RNA interference. It has been described recently that viruses can be transported over the BBB after osmotic disruption using mannitol. This superselective intra-arterial cerebral infusion technique is already in use for the treatment of patients with recurrent malignant glioma in a phase I study and would present an alternative way to suppress CHKα independent from pharmacological compounds [53, 54]. We were able to successfully monitor CHKα and EMT inhibition through reduced intracellular PC and tCho, both after genetic modification of the cells as well as after drug application using non-invasive ^1^H NMR spectroscopy. This *ex vivo* metabolic imaging technology raises the interesting option to potentially monitor the EMT-status in cells by quantifying their intracellular choline concentrations. *In vivo* proof of principle experiments are necessary to assess whether this concept might be applied for GBM diagnostics and therapeutic treatment surveillance.

The exact mechanism of CHKα-dependent suppression of EMT activators and reduction of GBM cell growth, invasiveness, and clonogenicity remains to be investigated. However, choline metabolism and especially CHKα activity has been correlated with malignant progression in various cancers [24, 32, 55, 56]. In the past, the RAS signaling pathway was found to regulate CHKα expression during tumor progression via phosphoinositide 3-kinase (PI3K) [55, 57, 58], thus directly linking CHKα activity to known oncogenes and signaling pathways driving tumor progression. Also EMT is regulated by Ras and PI3K signaling [59–61], making an indirect connection between choline metabolism and EMT regulation possible. Furthermore, a most recent study of Hu et al. described CHKα itself to be an important player in EGFR/PI3K/AKT signaling in colorectal cancer. Therefore, CHKα-mediated EMT induction via activation of EGFR/PI3K/AKT signaling could be a possible explanation for our observations but of course needs further investigations [62, 63]. As previous studies showed that CHKα overexpression increases cellular invasiveness, drug resistance and metastasis formation [25, 49], establishing a CHKα overexpression model in GBM would be a desirable way to further analyze the reciprocal regulation between choline metabolism and EMT in the future.

EMT activation in cancer cells is associated with increased chemotherapeutic resistance [64–66]. We found that combinatory treatment with the standard of care chemotherapeutic temozolomide (TMZ) and V-11-0711 causes synergistic cytotoxic effects in GBM1 cells (Figure 9). Our results in JHH520 cells were inconsistent, indicating that not the CHKα expression level itself regulates drug resistance and subsequent downstream analyses are needed to address the exact mechanism.

Besides the cholinic phenotype, we detected alterations of intracellular metabolites associated with other metabolic networks after EMT/CHKα inhibition such as increased creatine. Cre has been described as a putative anti-cancer agent and could be correlated with inhibition of tumor cell growth [67, 68]. The observed increase of Cre could result from reduced creatine kinase B (CKB) activity. CKB catalyzes the phosphorylation of creatine to phosphocreatine, which has been shown to promote tumorigenesis [69, 70]. Indeed, depletion of CHKα resulted in reduced mRNA expression of *CKB* and an increase of the Cre/PCre ratio (Supplementary Figure S2). These results further emphasize the oncogenic role of CHKα in cancer cells and indicate that the effect on cellular metabolism caused by CHKα-mediated EMT reduction is not only limited to choline homeostasis.

In conclusion we could identify CHKα as a powerful regulator of EMT in GBM cells. This opens the possibility to target chemotherapy resistant BTSCs through impairing their mesenchymal differentiation. Furthermore, we confirmed V-11-0711 as a potent CHKα and EMT inhibitor and suggest that our identified EMT-oncometabolic network may also be helpful to develop more tailored diagnostics monitoring the invasive properties of GBMs as well as surveilling the success of anti-EMT therapy. Given the importance of EMT for tumor progression and tumor stem cells in a variety of other tissue types, our discoveries could impact also other fields of oncology.

## MATERIALS AND METHODS

### Cell culture and V-11-0711 treatment

LN229 was purchased from American Tissue Culture Collection (Manassas, VA). JHH520 neurospheres were generously provided by G. Riggins (Johns Hopkins Hospital Baltimore, United States of America) and GBM1 neurospheres were generously provided by Dr Angelo Vescovi (Milan, Italy). HEK293T cells were also purchased from American Tissue Culture Collection (Manassas, VA). All cell lines were cultivated under standard cell culture conditions of temperature (37°C) and carbon dioxide (5%). GBM1 and JHH520 cell lines were both cultured as neurospheres in DMEM w/o pyruvate (Gibco) supplemented with 30% Ham's F12 Nutrient Mix (Gibco), 2% serum free B27 supplement (Gibco), 20 ng/ml human recombinant bFGF (Peprotech), 20 ng/ml human recombinant EGF (Peprotech), 5 μg/ml Heparin (Sigma Aldrich), and 1x Anti-Anti Penicillin Steptomycin Fungizone^®^ mixture (Gibco). LN229 and HEK293T cells were propagated to monolayer growth in DMEM with pyruvate (Gibco) plus 10% Fetal Calf Serum (FCS; Biochrome) and 1× Anti-Anti Penicillin Steptomycin Fungizone^®^ mixture (Gibco). Cells were passaged regularly to avoid acidification of media.

All cell lines were routinely tested for the absence of mycoplasma contamination using the PCR-based Mycoplasma Test Kit I/C from Promokine and tested for their identity using short tandem repeat testing [71].

A stock solution of the CHKα inhibitor V-11-0711 was prepared in DMSO and stored at −20°C. For immunoblotting, cells were cultured for 48 h under general cell culture conditions in the presence of various concentrations of V-11-0711 diluted in neurosphere medium. For all experiments with V-11-0711 we decided to use JHH520 and GBM1 cells as they both can be cultured without 10% serum which we found to interfere with the activity of small molecules.

For the combinatory treatment with TMZ and V-11-0711, the cells were cultured with different concentrations of the single drugs or both in combination (Single doses of V-11-0711: 0.47 μM for GBM1 and 0.2 μM for JHH520; Single doses of TMZ: 17.5 μM). Therefore, triplicates of 2000 cells in 100 μl were seeded in 96 –wells and cultured in medium supplemented with the desired drug concentration. After six days the cell viability was determined with the CellTiter-Blue^®^ Cell Viability Assay (Promega) due to the manufacturer's instructions. The combination index (CI) was calculated as described before using the program CompuSyn (ComboSyn Inc., Paramus, NJ. 07652 USA) [72]. Due to the algorithm of the software, a CI < 1 accounts for synergistic, a CI = 1 for additive, and a CI > 1 for antagonistic effects. The significance of the synergistic effect (*p < 0.05) was calculated compared to additive effect (CI = 1).

### Generation of lentiviral particles

The third generation lentiviral packaging system was used for the generation of lentiviral particles as previously described [10]. In brief, HEK293T cells were transfected with the lentiviral vector of choice and three different packaging plasmids (pMDLgpRRE, pRSVREV and pMD2VSVG) using GeneJuice^®^ Transfection Reagent (Merck Millipore). Virus supernatant was collected at time points 48 h, 72 h and 96 h post transfection. Interference RNA sequences against CHKα were designed with the software Primer3 [73] and cloned into the pLKO.1 TRC vector (Addgene plasmid #10878, [74]). Plasmids containing shRNAs against ZEB1 were derived as described previously [12].

### Quantitative real time PCR

Total RNA was extracted using the RNeasy Mini Kit (Qiagen) due to the manufacturer's instructions. The RNA concentration was photometrically assessed using the Nanodrop2000 spectrometer (Thermo Scientific). Two micrograms of RNA were utilized to synthesize complementary cDNA single strands using M-MLV reverse transcriptase (Promega) and random hexameric primers. Quantitative real time PCR was carried out using the Sso Advanced SYBR Green Supermix (BioRad) in a CFX Connect Thermocycler (BioRad). A total of 10 ng cDNA and 10 pmol per primer were used in each qPCR reaction. The relative quantifications were normalized to the endogenous housekeeping genes β-actin and β-2-microglobulin. Calculation of normalized relative gene expression was performed by supplied software of the CFX Connect Real-Time PCR Detection System (Bio-Rad). The figures show data from three independent experiments represented as mean ± SD. An unpaired student *t* test was performed to calculate statistical significance. The Primer sequences can be found in Supplementary Table S1.

### Western blotting

Cells were lysed in ice-cold RIPA buffer and protein concentrations were determined using the DC Protein Assay Kit (BioRad). Incubation with primary antibodies against ZEB1 (1:2000, Sigma #HPA027524), CHKα (1:500, Abcam #ab88053), TWIST1 (1:100, Santa Cruz #sc-81417), β-actin (1:1000, Santa Cruz #sc-130657) and α-tubulin (1:10000, Sigma #T9026) was performed overnight at 4°C on a 3D-shaker in 5% milk powder (Carl Roth) in TBST. As secondary antibodies we used goat-anti-rabbit IRDye800CW (1:10000, LI-COR #926-32211), goat-anti-mouse IRDye680RD (1:10000, LI-COR #926-68070) and goat anti-rabbit-HRP (1:10000, Jackson Immuno Research #111-035-144) diluted in blocking solution and incubated for 1 h at room temperature. Signal detection was performed either on a film based system by applying Super Signal West Pico Chemiluminescent Substrate (Thermo Scientific) or on a luminescence based system in a LI-COR Odyssey CLx Imager (LI-COR). Densitometry was done using supplied software from LI-COR or ImageJ software [75]. Densitometry values for ZEB1 and CHKα were normalized to the corresponding alpha tubulin values, TWIST1 was normalized to beta actin.

### Dual-phase metabolite extraction of cell cultures

At least 5 × 10^6^ cells were harvested, counted in triplicates and subjected to methanol/chloroform/water (1:1:1, v/v/v) dual-phase extraction as previously described [22, 76]. In brief, cells were washed twice with 10 ml ice-cold saline, resuspended in 850 μl ice-cold ddH_2_O and transferred into a prechilled glass centrifuge tube. A total of 4 ml of ice-cold methanol were added and the cells were vortexed vigorously and incubated on ice for 15 min. Then 4 ml of ice-cold chloroform were added, vortexed and incubated for 10 min on ice. Finally, 3.15 ml of chilled ddH_2_O were added, vortexed and incubated at 4°C overnight to enable phase separation. Next day, the samples were centrifuged for 30 min at 4500 rpm at 4°C, and the upper phase containing the water soluble metabolites was carefully separated, supplemented with 10 mg of Chelex^®^ 100 resin (Sigma Aldrich) and incubated on ice for 10 min to remove divalent cations. After filtration through a 70 μm mesh the samples were centrifuged for 1 h in a vacuum concentrator at 30°C to evaporate the methanol. Subsequently, the samples were frozen at −80°C, lyophilized and stored at −80°C until measurement.

### ^1^H NMR data acquisition and processing

The lyophilisates were resuspended in 20 mM phosphate buffer (pH 7.0) containing 10% D_2_O and 4,4-dimethyl-4-silapentane-1-sulfonic acid (DSS; euriso-top) or 3-(Trimethylsilyl) propanoic acid (TSP; Lancaster Synthesis) as an internal standard.

^1^H NMR spectra of extracts were acquired on a Bruker AVANCE III HD 700 spectrometer equipped with a 5 mm HCN TCI cryo-probe operating at 700 MHz (16.4 Tesla). The ^1^H-NMR data were obtained using excitation sculpting for water suppressing and the following acquisition parameters: 25°C sample temperature, 9800 Hz sweep width, 3.2 s repetition time and time-domain data points of 32 K and 256 transients.

Spectra were processed and analyzed using Mestrenova version 8.0.1-10878 (Mestrelab Research .L.). Metabolite intensities of different samples were normalized to a standard of the same concentration in each measurement. The figures show data from three independent experiments represented as mean ± SD. Furthermore, an unpaired student *t* test was performed to calculate statistical significance.

### Double immunofluorescence stainings

LN229, GBM1, and JHH529 cells were plated onto coverslips in 24-well plates. For GBM1 and JHH520 cells, the coverslips were pre-coated with 50 μg/ml laminin (Sigma) for 1 h at 37°C. After three hours incubation at 37°C and 5% CO_2_, the cells were washed with PBS and fixed with 4% Paraformaldehyde in PBS for 20 min at RT. The cells were washed again with PBS and incubated with blocking buffer (PBS pH 7.4, 10% Normal Goat Serum (Gibco), 0.5% TX-100, 0.05% Tween20) for 2 h at RT. Cells were stained with rabbit-anti-CHKα (1:250, Abcam # ab88053) and either mouse-anti-VIMENTIN (1:1000, #) or mouse-anti-SOX2 (1:100, Cell Signaling #4900S) primary antibodies diluted in blocking buffer overnight at 4°C. Secondary antibodies (goat anti-mouse Alexafluor488 (1:1000, Thermo Fisher #A-11029) and goat anti-rabbit Alexafluor594 (1:1000, Thermo Fisher #A-11037)) were incubated for 2 h at RT. Preparations were mounted in ProLong Gold + DAPI (Thermo Fisher) and fluorescent images were obtained by a LSM 700 microscope, Carl Zeiss, and analyzed in ZEN software (Carl Zeiss). Controls were performed with omission of one or both primary antibodies.

### Cell viability and apoptosis assays

For the assessment of cell viability, cell numbers were adjusted to defined concentrations (20.000 cells/ml for GBM1 and JHH520; 15.000 cells/ml for LN229). For the analysis of ZEB1 and CHKα knockdown cells we used cells from passages 3 to 10 post transduction to exclude a transient effect from viral transduction. Triplicates of 100 μl were plated into each well of a 96-well plate. On five consecutive days the relative viable cell mass was determined using the CellTiter-Blue^®^ Cell Viability Assay (Promega) due to the manufacturer's instructions. Fluorescence was measured after two h substrate incubation on cells using the Tecan Safire 2 Multiplate reader (Tecan) at 560ex/590em. Exponential growth curves were calculated for each condition and displayed in the graphs with GraphPad Prism 5 (GraphPad Software, Inc., USA).

In order to test if V-11-0711 treatment induces apoptosis in GBM cells, GBM1 and JHH520 cells were treated with different concentrations of V-11-0711 for 48 h. Afterwards, the percentages of living, early apoptotic, late apoptotic, and dead cells were assessed using the “Muse^®^ Annexin V and Dead Cell Assay Kit” for the Muse^®^ cell analyzer (Merck Millipore) due to the manufacturer's instructions.

### Invasion and clonogenicity assays

Invasion of GBM cells was assessed with a modified Boyden chamber assay as described previously [12]. In brief, a total of 1*10^5^ cells were plated per Matrigel (BD) coated insert in DMEM w/o FCS, DMEM with 10% FCS was added in the lower chamber. After an incubation of 24 h the experiment was terminated and the remaining non-invaded cells on the upper surface of the membrane were removed carefully by swabbing. The filter was fixed with methanol (−20°C) for 10 min, washed with PBS and subsequently stained with hematoxylin for 5 min. After destaining with warm water, five pictures were taken per well and the stained invaded cells were counted. For the drug treatment experiments, cells were pretreated for 24 h with 1 μM (GBM1) or 0.5 μM (JHH520) V-11-0711 in standard culture conditions before assessing their invasiveness in a time window of 24 h.

For the assessment of the clonogenic capacity of GBM cells we performed Soft agar assays as described previously [71]. In brief, six-well plates were coated with 1.5 ml of a base layer of 1% agarose (Gibco) in neurosphere medium and placed in 4°C for 1 h. A top layer containing 0.6% agarose and a single-cell suspension (3500 cells/well for GBM1 and 5000 cells/well for JHH520) in 2 ml neurosphere medium was plated on top of the base layer and incubated at RT for 1 h. Once the cell layer was solidified 2 ml of neurosphere medium (for V-11-0711 treatment studies either supplemented with drug or vehicle) were added to each well. Every three days 500 μl fresh medium (for V-11-0711 treatment studies either supplemented with drug or vehicle) were added to each well. On day 21, 1 ml of a 1 mg/ml 4-Nitro blue tetrazolium chloride (NBT) solution (Sigma Aldrich) was added and incubated overnight at 37°C to stain the colonies. The experiments were quantified using the Clono Counter software [77].

### RNA sequencing data from IVY Glioblastoma Project

RNA sequencing data was generated from anatomic structures isolated by laser microdissection. Five tumor structures (Leading Edge, Infiltrating Tumor, Cellular Tumor, Microvascular Proliferation, and Pseudopalisading Cells around Necrosis) were identified by H&E staining and compared to hyperplastic blood vessels and the microvascular proliferative region. A total of 122 RNA samples were generated from 10 tumors and used for sequencing. Website: © 2015 Allen Institute for Brain Science. Ivy Glioblastoma Atlas Project [Internet]. Available from: glioblastoma.alleninstitute.org.

## SUPPLEMENTARY MATERIALS FIGURES AND TABLE



## References

[1] Stupp R, Hegi ME, Mason WP, van den Bent MJ, Taphoorn MJ, Janzer RC, Ludwin SK, Allgeier A, Fisher B, Belanger K, Hau P, Brandes AA, Gijtenbeek J (2009). Effects of radiotherapy with concomitant and adjuvant temozolomide versus radiotherapy alone on survival in glioblastoma in a randomised phase III study: 5-year analysis of the EORTC-NCIC trial. The Lancet Oncology.

[2] Tabatabai G, Stupp R, van den Bent MJ, Hegi ME, Tonn JC, Wick W, Weller M (2010). Molecular diagnostics of gliomas: the clinical perspective. Acta neuropathologica.

[3] Furnari FB, Fenton T, Bachoo RM, Mukasa A, Stommel JM, Stegh A, Hahn WC, Ligon KL, Louis DN, Brennan C, Chin L, DePinho RA, Cavenee WK (2007). Malignant astrocytic glioma: genetics, biology, and paths to treatment. Genes & development.

[4] Singh SK, Hawkins C, Clarke ID, Squire JA, Bayani J, Hide T, Henkelman RM, Cusimano MD, Dirks PB (2004). Identification of human brain tumour initiating cells. Nature.

[5] Zhou BB, Zhang H, Damelin M, Geles KG, Grindley JC, Dirks PB (2009). Tumour-initiating cells: challenges and opportunities for anticancer drug discovery. Nature reviews Drug discovery.

[6] Liu G, Yuan X, Zeng Z, Tunici P, Ng H, Abdulkadir IR, Lu L, Irvin D, Black KL, Yu JS (2006). Analysis of gene expression and chemoresistance of CD133+ cancer stem cells in glioblastoma. Molecular cancer.

[7] Bao S, Wu Q, McLendon RE, Hao Y, Shi Q, Hjelmeland AB, Dewhirst MW, Bigner DD, Rich JN (2006). Glioma stem cells promote radioresistance by preferential activation of the DNA damage response. Nature.

[8] Mani SA, Guo W, Liao MJ, Eaton EN, Ayyanan A, Zhou AY, Brooks M, Reinhard F, Zhang CC, Shipitsin M, Campbell LL, Polyak K, Brisken C (2008). The epithelial-mesenchymal transition generates cells with properties of stem cells. Cell.

[9] Scheel C, Weinberg RA (2012). Cancer stem cells and epithelial-mesenchymal transition: concepts and molecular links. Seminars in cancer biology.

[10] Kahlert UD, Maciaczyk D, Doostkam S, Orr BA, Simons B, Bogiel T, Reithmeier T, Prinz M, Schubert J, Niedermann G, Brabletz T, Eberhart CG, Nikkhah G (2012). Activation of canonical WNT/beta-catenin signaling enhances *in vitro* motility of glioblastoma cells by activation of ZEB1 and other activators of epithelial-to-mesenchymal transition. Cancer letters.

[11] Siebzehnrubl FA, Silver DJ, Tugertimur B, Deleyrolle LP, Siebzehnrubl D, Sarkisian MR, Devers KG, Yachnis AT, Kupper MD, Neal D, Nabilsi NH, Kladde MP, Suslov O (2013). The ZEB1 pathway links glioblastoma initiation, invasion and chemoresistance. EMBO molecular medicine.

[12] Kahlert UD, Suwala AK, Raabe EH, Siebzehnrubl FA, Suarez MJ, Orr BA, Bar EE, Maciaczyk J, Eberhart CG (2015). ZEB1 Promotes Invasion in Human Fetal Neural Stem Cells and Hypoxic Glioma Neurospheres. Brain pathology.

[13] Elias MC, Tozer KR, Silber JR, Mikheeva S, Deng M, Morrison RS, Manning TC, Silbergeld DL, Glackin CA, Reh TA, Rostomily RC (2005). TWIST is expressed in human gliomas and promotes invasion. Neoplasia.

[14] Han SP, Kim JH, Han ME, Sim HE, Kim KS, Yoon S, Baek SY, Kim BS, Oh SO (2011). SNAI1 is involved in the proliferation and migration of glioblastoma cells. Cellular and molecular neurobiology.

[15] Praveen Kumar VR, Sehgal P, Thota B, Patil S, Santosh V, Kondaiah P (2014). Insulin like growth factor binding protein 4 promotes GBM progression and regulates key factors involved in EMT and invasion. Journal of neuro-oncology.

[16] Lin JJ, Zhao TZ, Cai WK, Yang YX, Sun C, Zhang Z, Xu YQ, Chang T, Li ZY (2015). Inhibition of histamine receptor 3 suppresses glioblastoma tumor growth, invasion, and epithelial-to-mesenchymal transition. Oncotarget.

[17] Cai JJ, Qi ZX, Chen LC, Yao Y, Gong Y, Mao Y (2016). miR-124 suppresses the migration and invasion of glioma cells *in vitro* via Capn4. Oncology reports.

[18] Sanchez-Martinez R, Cruz-Gil S, Gomez de Cedron M, Alvarez-Fernandez M, Vargas T, Molina S, Garcia B, Herranz J, Moreno-Rubio J, Reglero G, Perez-Moreno M, Feliu J, Malumbres M (2015). A link between lipid metabolism and epithelial-mesenchymal transition provides a target for colon cancer therapy. Oncotarget.

[19] Griffin JL, Shockcor JP (2004). Metabolic profiles of cancer cells. Nature reviews Cancer.

[20] Cairns RA, Harris IS, Mak TW (2011). Regulation of cancer cell metabolism. Nature reviews Cancer.

[21] Warburg O (1956). On the origin of cancer cells. Science.

[22] Glunde K, Jie C, Bhujwalla ZM (2004). Molecular causes of the aberrant choline phospholipid metabolism in breast cancer. Cancer research.

[23] Granata A, Nicoletti R, Tinaglia V, De Cecco L, Pisanu ME, Ricci A, Podo F, Canevari S, Iorio E, Bagnoli M, Mezzanzanica D (2014). Choline kinase-alpha by regulating cell aggressiveness and drug sensitivity is a potential druggable target for ovarian cancer. British journal of cancer.

[24] Hernando E, Sarmentero-Estrada J, Koppie T, Belda-Iniesta C, Ramirez de Molina V, Cejas P, Ozu C, Le C, Sanchez JJ, Gonzalez-Baron M, Koutcher J, Cordon-Cardo C, Bochner BH (2009). A critical role for choline kinase-alpha in the aggressiveness of bladder carcinomas. Oncogene.

[25] Hu L, Wang RY, Cai J, Feng D, Yang GZ, Xu QG, Zhai YX, Zhang Y, Zhou WP, Cai QP (2016). Overexpression of CHKΑ contributes to tumor progression and metastasis and predicts poor prognosis in colorectal carcinoma. Oncotarget.

[26] Aboagye EO, Bhujwalla ZM (1999). Malignant transformation alters membrane choline phospholipid metabolism of human mammary epithelial cells. Cancer research.

[27] Ackerstaff E, Glunde K, Bhujwalla ZM (2003). Choline phospholipid metabolism: a target in cancer cells?. Journal of cellular biochemistry.

[28] Glunde K, Bhujwalla ZM, Ronen SM (2011). Choline metabolism in malignant transformation. Nature reviews Cancer.

[29] Herminghaus S, Pilatus U, Moller-Hartmann W, Raab P, Lanfermann H, Schlote W, Zanella FE (2002). Increased choline levels coincide with enhanced proliferative activity of human neuroepithelial brain tumors. NMR in biomedicine.

[30] Ramirez de Molina A, Gallego-Ortega D, Sarmentero-Estrada J, Lagares D, Gomez Del Pulgar T, Bandres E, Garcia-Foncillas J, Lacal JC (2008). Choline kinase as a link connecting phospholipid metabolism and cell cycle regulation: implications in cancer therapy. The international journal of biochemistry & cell biology.

[31] Gruber J, See Too WC, Wong MT, Lavie A, McSorley T, Konrad M (2012). Balance of human choline kinase isoforms is critical for cell cycle regulation: implications for the development of choline kinase-targeted cancer therapy. The FEBS journal.

[32] Falcon SC, Hudson CS, Huang Y, Mortimore M, Golec JM, Charlton PA, Weber P, Sundaram H (2013). A non-catalytic role of choline kinase alpha is important in promoting cancer cell survival. Oncogenesis.

[33] Mori N, Wildes F, Kakkad S, Jacob D, Solaiyappan M, Glunde K, Bhujwalla ZM (2015). Choline kinase-alpha protein and phosphatidylcholine but not phosphocholine are required for breast cancer cell survival. NMR in biomedicine.

[34] Wang Y, Wen M, Kwon Y, Xu Y, Liu Y, Zhang P, He X, Wang Q, Huang Y, Jen KY, LaBarge MA, You L, Kogan SC (2014). CUL4A induces epithelial-mesenchymal transition and promotes cancer metastasis by regulating ZEB1 expression. Cancer research.

[35] Sintov E, Nathan G, Knoller S, Pasmanik-Chor M, Russ HA, Efrat S (2015). Inhibition of ZEB1 expression induces redifferentiation of adult human beta cells expanded *in vitro*. Scientific reports.

[36] Liu Y, El-Naggar S, Darling DS, Higashi Y, Dean DC (2008). Zeb1 links epithelial-mesenchymal transition and cellular senescence. Development.

[37] Hugo HJ, Pereira L, Suryadinata R, Drabsch Y, Gonda TJ, Gunasinghe NP, Pinto C, Soo ET, van Denderen BJ, Hill P, Ramsay RG, Sarcevic B, Newgreen DF (2013). Direct repression of MYB by ZEB1 suppresses proliferation and epithelial gene expression during epithelial-to-mesenchymal transition of breast cancer cells. Breast cancer research.

[38] Wellner U, Schubert J, Burk UC, Schmalhofer O, Zhu F, Sonntag A, Waldvogel B, Vannier C, Darling D, zur Hausen A, Brunton VG, Morton J, Sansom O (2009). The EMT-activator ZEB1 promotes tumorigenicity by repressing stemness-inhibiting microRNAs. Nature cell biology.

[39] Furse S, de Kroon AI (2015). Phosphatidylcholine's functions beyond that of a membrane brick. Molecular membrane biology.

[40] Cazzolli R, Shemon AN, Fang MQ, Hughes WE (2006). Phospholipid signalling through phospholipase D and phosphatidic acid. IUBMB life.

[41] Gangemi RM, Griffero F, Marubbi D, Perera M, Capra MC, Malatesta P, Ravetti GL, Zona GL, Daga A, Corte G (2009). SOX2 silencing in glioblastoma tumor-initiating cells causes stop of proliferation and loss of tumorigenicity. Stem cells.

[42] Favaro R, Valotta M, Ferri AL, Latorre E, Mariani J, Giachino C, Lancini C, Tosetti V, Ottolenghi S, Taylor V, Nicolis SK (2009). Hippocampal development and neural stem cell maintenance require Sox2-dependent regulation of Shh. Nature neuroscience.

[43] Mikheeva SA, Mikheev AM, Petit A, Beyer R, Oxford RG, Khorasani L, Maxwell JP, Glackin CA, Wakimoto H, Gonzalez-Herrero I, Sanchez-Garcia I, Silber JR, Horner PJ (2010). TWIST1 promotes invasion through mesenchymal change in human glioblastoma. Molecular cancer.

[44] Pang H, Zheng Y, Zhao Y, Xiu X, Wang J (2015). miR-590-3p suppresses cancer cell migration, invasion and epithelial-mesenchymal transition in glioblastoma multiforme by targeting ZEB1 and ZEB2. Biochemical and biophysical research communications.

[45] Rahme GJ, Israel MA (2015). Id4 suppresses MMP2-mediated invasion of glioblastoma-derived cells by direct inactivation of Twist1 function. Oncogene.

[46] Wang N, Guo D, Zhao YY, Dong CY, Liu XY, Yang BX, Wang SW, Wang L, Liu QG, Ren Q, Lin YM, Ma XT (2015). TWIST-1 promotes cell growth, drug resistance and progenitor clonogenic capacities in myeloid leukemia and is a novel poor prognostic factor in acute myeloid leukemia. Oncotarget.

[47] Liu H, Wang H, Liu X, Yu T (2016). miR-1271 inhibits migration, invasion and epithelial-mesenchymal transition by targeting ZEB1 and TWIST1 in pancreatic cancer cells. Biochemical and biophysical research communications.

[48] Dou J, He X, Liu Y, Wang Y, Zhao F, Wang X, Chen D, Shi F, Wang J (2014). Effect of downregulation of ZEB1 on vimentin expression, tumour migration and tumourigenicity of melanoma B16F10 cells and CSCs. Cell biology international.

[49] Shah T, Wildes F, Penet MF, Winnard PT, Glunde K, Artemov D, Ackerstaff E, Gimi B, Kakkad S, Raman V, Bhujwalla ZM (2010). Choline kinase overexpression increases invasiveness and drug resistance of human breast cancer cells. NMR in biomedicine.

[50] Dahlrot RH, Hermansen SK, Hansen S, Kristensen BW (2013). What is the clinical value of cancer stem cell markers in gliomas?. International journal of clinical and experimental pathology.

[51] Singh SK, Clarke ID, Terasaki M, Bonn VE, Hawkins C, Squire J, Dirks PB (2003). Identification of a cancer stem cell in human brain tumors. Cancer research.

[52] Kahlert UD, Bender NO, Maciaczyk D, Bogiel T, Bar EE, Eberhart CG, Nikkhah G, Maciaczyk J (2012). CD133/CD15 defines distinct cell subpopulations with differential *in vitro* clonogenic activity and stem cell-related gene expression profile in *in vitro* propagated glioblastoma multiforme-derived cell line with a PNET-like component. Folia neuropathologica.

[53] Foley CP, Rubin DG, Santillan A, Sondhi D, Dyke JP, Gobin YP, Crystal RG, Ballon DJ (2014). Intra-arterial delivery of AAV vectors to the mouse brain after mannitol mediated blood brain barrier disruption. Journal of controlled release.

[54] Janowski M, Walczak P, Pearl MS (2016). Predicting and optimizing the territory of blood-brain barrier opening by superselective intra-arterial cerebral infusion under dynamic susceptibility contrast MRI guidance. Journal of cerebral blood flow and metabolism.

[55] Janardhan S, Srivani P, Sastry GN (2006). Choline kinase: an important target for cancer. Current medicinal chemistry.

[56] Tedeschi G, Lundbom N, Raman R, Bonavita S, Duyn JH, Alger JR, Di Chiro G (1997). Increased choline signal coinciding with malignant degeneration of cerebral gliomas: a serial proton magnetic resonance spectroscopy imaging study. Journal of neurosurgery.

[57] Ramirez de Molina A, Penalva V, Lucas L, Lacal JC (2002). Regulation of choline kinase activity by Ras proteins involves Ral-GDS and PI3K. Oncogene.

[58] Ramirez de Molina A, Rodriguez-Gonzalez A, Penalva V, Lucas L, Lacal JC (2001). Inhibition of ChoK is an efficient antitumor strategy for Harvey-, Kirsten-, and N-ras-transformed cells. Biochemical and biophysical research communications.

[59] Wu K, Fan J, Zhang L, Ning Z, Zeng J, Zhou J, Li L, Chen Y, Zhang T, Wang X, Hsieh JT, He D (2012). PI3K/Akt to GSK3beta/beta-catenin signaling cascade coordinates cell colonization for bladder cancer bone metastasis through regulating ZEB1 transcription. Cellular signalling.

[60] Tse JC, Kalluri R (2007). Mechanisms of metastasis: epithelial-to-mesenchymal transition and contribution of tumor microenvironment. Journal of cellular biochemistry.

[61] Saitoh M, Endo K, Furuya S, Minami M, Fukasawa A, Imamura T, Miyazawa K (2016). STAT3 integrates cooperative Ras and TGF-beta signals that induce Snail expression. Oncogene.

[62] Zhong Z, Hu Z, Jiang Y, Sun R, Chen X, Chu H, Zeng M, Sun C (2016). Interleukin-11 promotes epithelial-mesenchymal transition in anaplastic thyroid carcinoma cells through PI3K/Akt/GSK3beta signaling pathway activation. Oncotarget.

[63] Cheng JC, Auersperg N, Leung PC (2012). EGF-induced EMT and invasiveness in serous borderline ovarian tumor cells: a possible step in the transition to low-grade serous carcinoma cells?. PloS one.

[64] Shang Y, Cai X, Fan D (2013). Roles of epithelial-mesenchymal transition in cancer drug resistance. Current cancer drug targets.

[65] Singh A, Settleman J (2010). EMT, cancer stem cells and drug resistance: an emerging axis of evil in the war on cancer. Oncogene.

[66] Sui H, Zhu L, Deng W, Li Q (2014). Epithelial-mesenchymal transition and drug resistance: role, molecular mechanisms, and therapeutic strategies. Oncology research and treatment.

[67] Miller EE, Evans AE, Cohn M (1993). Inhibition of rate of tumor growth by creatine and cyclocreatine.

[68] Campos-Ferraz PL, Gualano B, das Neves W, Andrade IT, Hangai I, Pereira RT, Bezerra RN, Deminice R, Seelaender M, Lancha AH (2016). Exploratory studies of the potential anti-cancer effects of creatine. Amino acids.

[69] Wallimann T, Tokarska-Schlattner M, Schlattner U (2011). The creatine kinase system and pleiotropic effects of creatine. Amino acids.

[70] Loo JM, Scherl A, Nguyen A, Man FY, Weinberg E, Zeng Z, Saltz L, Paty PB, Tavazoie SF (2015). Extracellular metabolic energetics can promote cancer progression. Cell.

[71] Kahlert UD, Suwala AK, Koch K, Natsumeda M, Orr BA, Hayashi M, Maciaczyk J, Eberhart CG (2015). Pharmacologic Wnt Inhibition Reduces Proliferation, Survival, and Clonogenicity of Glioblastoma Cells. Journal of neuropathology and experimental neurology.

[72] Martin KJ, Chen SF, Clark GM, Degen D, Wajima M, Von Hoff DD, Kaddurah-Daouk R (1994). Evaluation of creatine analogues as a new class of anticancer agents using freshly explanted human tumor cells. Journal of the National Cancer Institute.

[73] Untergasser A, Cutcutache I, Koressaar T, Ye J, Faircloth BC, Remm M, Rozen SG (2012). Primer3—new capabilities and interfaces. Nucleic acids research.

[74] Moffat J, Grueneberg DA, Yang X, Kim SY, Kloepfer AM, Hinkle G, Piqani B, Eisenhaure TM, Luo B, Grenier JK, Carpenter AE, Foo SY, Stewart SA (2006). A lentiviral RNAi library for human and mouse genes applied to an arrayed viral high-content screen. Cell.

[75] Schneider CA, Rasband WS, Eliceiri KW (2012). NIH Image to ImageJ: 25 years of image analysis. Nature methods.

[76] Tyagi RK, Azrad A, Degani H, Salomon Y (1996). Simultaneous extraction of cellular lipids and water-soluble metabolites: evaluation by NMR spectroscopy. Magnetic resonance in medicine.

[77] Niyazi M, Niyazi I, Belka C (2007). Counting colonies of clonogenic assays by using densitometric software. Radiation oncology.

