# Supplementary material for: Reciprocal regulation of the cholinic phenotype and epithelial-mesenchymal transition in glioblastoma cells
Source: Oncotarget. 2016 Sep 29;7(45):73414–31. doi: 10.18632/oncotarget.12337 (PMC5341988; doi:10.18632/oncotarget.12337)
Supplement: Supplementary file 1 [file oncotarget-07-73414-s001.pdf]

Reciprocal regulation of the cholinic phenotype and epithelial-mesenchymal transition in glioblastoma cells

Supplementary Materials

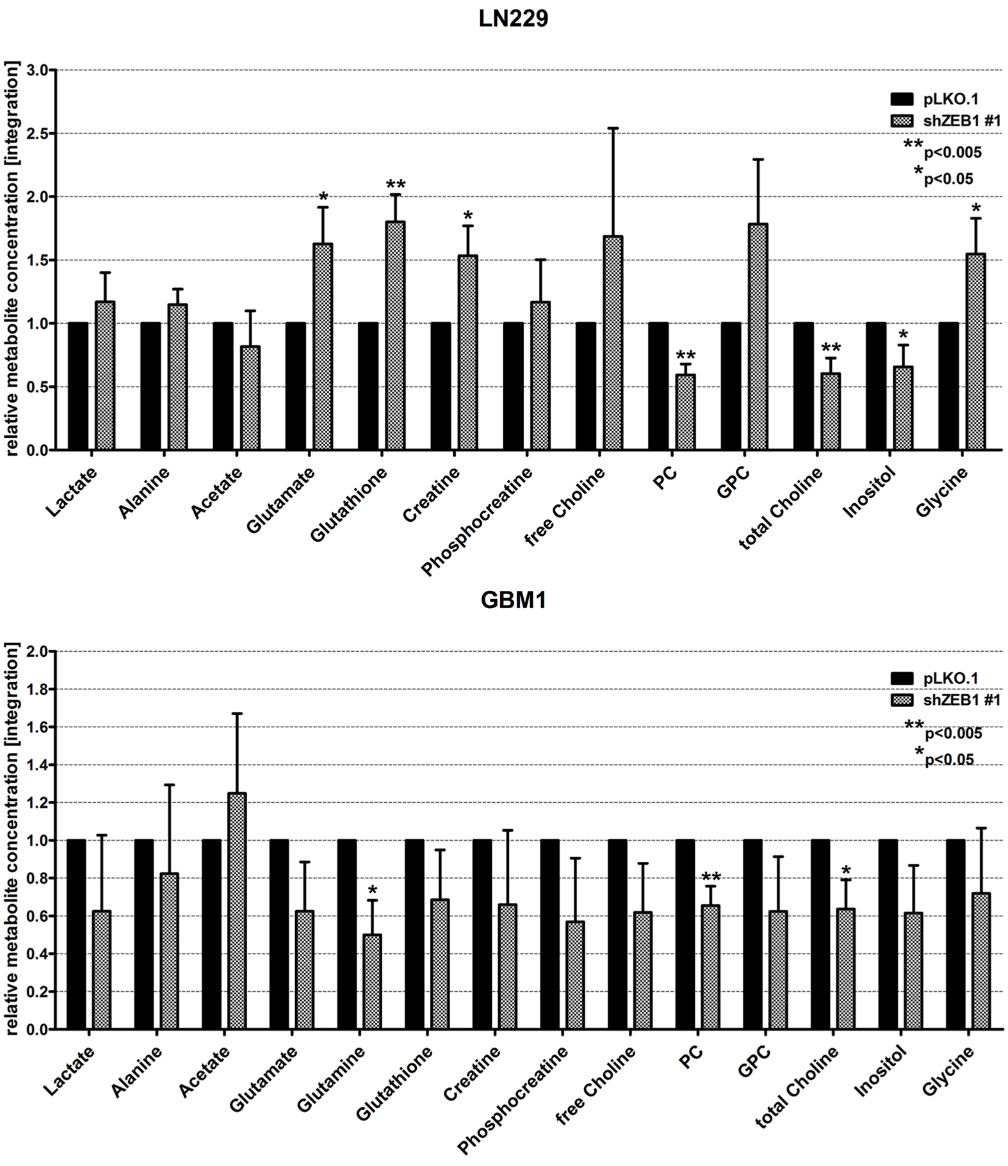

Supplementary Figure S1: ZEB1 alters the metabolism of LN229 and GBM1 cells. Water soluble metabolites of shZEB1#1 and pLKO.1 control cells were analyzed with 1H-NMR spectroscopy. The relative metabolite concentrations of three independent experiments are displayed as mean ± SD.

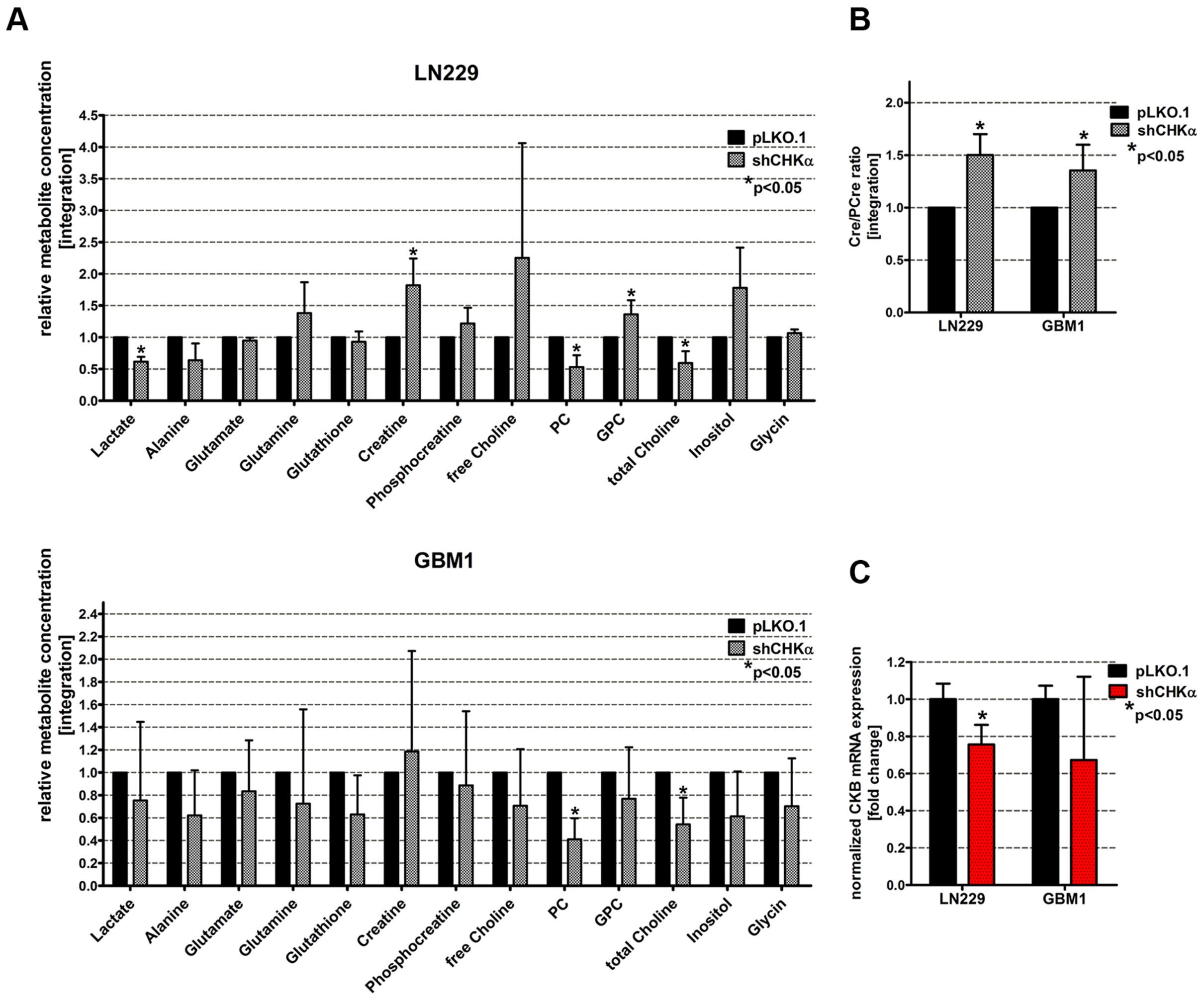

Supplementary Figure S2: CHKα knockdown alters the metabolism of LN229 and GBM1 cells. (**A**) Water soluble metabolites of shCHKα and pLKO.1 control cells were analyzed with 1H-NMR spectroscopy. (**B**) The ratio of the relative creatine and phosphocreatine concentrations were calculated from ^{1}H-NMR spectra of shCHKα and pLKO.1 control cells. (**C**) CHKα knockdown decreases the expression of creatine kinase brain-type (CKB), the enzyme phosphorylating creatine. The data is represented as mean ± SD (*n* = 3).

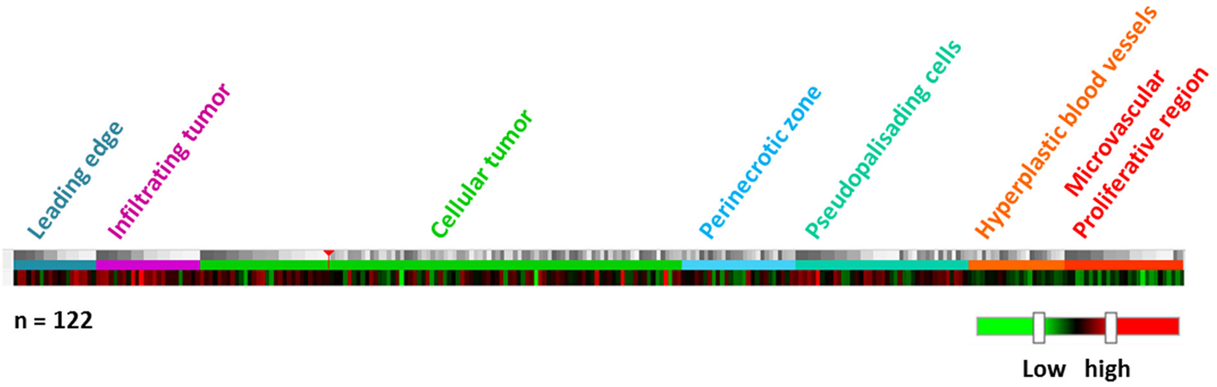

Supplementary Figure S3: CHKα is expressed in higher levels in the infiltrating zone and the leading edge of GBM. RNA sequencing data generated from anatomic structures isolated by laser microdissection. A total of 122 RNA samples were generated from 10 tumors and used for sequencing. Data from the IVY Glioblastoma project (Website: © 2015 Allen Institute for Brain Science. Ivy Glioblastoma Atlas Project [Internet]. Available from: glioblastoma.alleninstitute.org). Image credit: Allen Institute.

Supplementary Table S1: Primer sequences used in RT qPCR

Name	Gene name	Gene ID (NCBI)	Forward primer	Reverse primer
**β actin**	*ACTB*	60	CCCAGCACAATGAAGATCAA	CGATCCACACGGAGTACTTG
**β-2-microglobulin**	*B2M*	567	GTTGCTCCACAGGTAGCT CTAG	ACAAGCTTTGAGTGCAAGA GATTG
**CHKα**	*CHKA*	1119	GAAAGTGCTCCTGCGGCT GTATG	CGGCTCGGGATGAACTGCTC
**CKB**	*CKB*	1152	GGCAACATGAAGGAGGTGTT	ATGGGCAGGTGAGGATGTAG
**Nestin**	*NES*	10763	GGCGCACCTCAAGATGTCC	CTTGGGGTCCTGAAAGCTG
**N-cadherin**	*CDH2*	1000	TATGCCCAAGACAAAGAGACC	CAACTTCTGCTGACTCCTTCA
**SOX2**	*SOX2*	6657	TGGACAGTTACGCGCACA	CGAGTAGGACATGCTGTA
**SNAI1**	*SNAI1*	6615	GCTGCAGGACTCTAATCC	ATCTCCGGAGGTGGGATC
**SNAI2**	*SNAI2*	6591	TGGTTGCTTCAAGGACACAT	GTTGCAGTGAGGGCAAGAA
**TWIST1**	*TWIST1*	7291	TCCGCGTCCCACTAGCA	TTCTCTGGAAACAATGAC ATCTAGGT
**VIMENTIN**	*VIM*	7431	CCCTCACCTGTGAAGTGGAT	TCCAGCAGCTTCCTGTAGGT
**ZEB1**	*ZEB1*	6935	AAGAATTCACAGTGGAG AGAAGCCA	CGTTTCTTGCAGTTTGGGCATT
**ZEB2**	*ZEB2*	9839	GCCGCGGCATATGGTGACA	GCCACACTCTGTGCATTTGAA
